# Inferring Geographic Spread of Flaviviruses Through Analysis of Hypervariable Genomic Regions

**DOI:** 10.3390/tropicalmed10100277

**Published:** 2025-09-24

**Authors:** Jimena Sánchez-Nava, Mario H. Rodríguez, Eduardo D. Rodríguez-Aguilar

**Affiliations:** Centre for Research in Infectious Diseases, National Institute of Public Health, Av. Universidad 655, Cuernavaca 62100, Mexico; jimena.sn16@gmail.com (J.S.-N.); mhenry@insp.mx (M.H.R.)

**Keywords:** flavivirus, dengue virus, yellow fever virus, West Nile virus, Zika virus, phylogenetic reconstruction, geographic distribution

## Abstract

The Flaviviruses Dengue virus (DENV), West Nile virus (WNV), Zika virus (ZIKV), and Yellow Fever virus (YFV), are mosquito-borne viruses that represent a persistent challenge to global health due to the emergence and re-emergence of outbreaks of significant magnitudes. Their positive-sense RNA genome, about 11,000 nucleotides long, encodes structural and nonstructural proteins. These viruses evolve rapidly through mutations and genetic recombination, which can lead to more virulent and transmissible strains. Although whole-genome sequencing is ideal for studying their evolution and geographic spread, its cost is a limitation. We investigated the genetic variability of DENV, ZIKV, WNV, and YFV to identify genomic regions that accurately reflect the phylogeny of the complete coding sequence and evaluated the utility of these regions in reconstructing the geographic dispersal patterns of viral genotypes and lineages. Publicly available sequences from GenBank were examined to assess variability, reconstruct phylogenies, and identify the most informative genomic regions. Once representative regions were identified, they were used to infer the global phylogeographic structure of each virus. The virus depicted distinct variation patterns, but conserved regions of high and low variability were common to all. Highly variable regions of ~2700 nt offered greater resolution in phylogenetic trees, improving the definition of internal branches and statistical support for nodes. In some cases, combined multiple highly variable regions enhanced phylogenetic accuracy. Phylogeographic reconstruction consistently grouped sequences by genotype and geographic origin, with temporal structuring revealing evolutionarily distinct clusters that diverged over decades. These findings highlight the value of targeting genomic regions for phylogenetic and phylogeographic analysis, providing an efficient alternative for genomic surveillance.

## 1. Introduction

The Flavivirus genus comprises approximately 70 recognized members, several of which are pathogenic to humans, including dengue virus (DENV), yellow fever virus (YFV), West Nile virus (WNV), and Zika virus (ZIKV). These viruses are transmitted to humans through mosquito vectors [1]. Flavivirus infections can manifest in a variety of clinical symptoms, ranging from nonspecific febrile illnesses to more severe conditions like central nervous system infections, and acute hemorrhagic fever [2].

Flaviviruses possess a positive-sense, single-stranded RNA genome approximately 9000–12,500 nucleotides (nt) in length. Their genomes encode three structural proteins (C, M, and E) and seven nonstructural proteins (NS1, NS2A, NS2B, NS3, NS4A, NS4B, and NS5) [1]. A key characteristic of RNA viruses is their high mutation rate, which is substantially higher than that of DNA viruses due to the lack of proofreading activity of their RNA-dependent RNA polymerase [3]. While mutations arise randomly throughout the genome, viral genes contain conserved regions essential for structure and function, alongside more permissive regions that facilitate immune evasion through antigenic variation [4].

High mutation rates are the primary driver of genetic variability within Flavivirus populations, leading to continuous viral evolution and adaptation [5]. They estimated mutation rates range from 4.5 × 10^−3^ to 4 × 10^−4^ substitutions per site per year [6,7,8,9,10]. The accumulation of genetic changes generates structured genetic diversity within species, often observable as distinct genotypes and lineages with varying geographic distributions and epidemiological patterns. For instance, YFV presents at least four major genotypes across Africa and South America [11,12]; ZIKV includes West African, East African, and Asian genotypes [13,14]; and WNV has diversified into nine proposed lineages distributed across most continents [15,16]. DENV, with four serotypes (DENV-1 to DENV-4), is further subdivided into multiple genotypes within each serotype and circulates endemically throughout Southeast Asia, the Western Pacific, the Americas, and Africa [17,18].

Phylogenetic analysis, which reconstructs the evolutionary history and relationships among viral isolates, is a fundamental tool in molecular epidemiology. However, the accuracy of phylogenetic reconstruction depends on the rate of genetic change within the analyzed sequences; using regions with either few or an excess of mutations can reduce the accuracy of the analysis [19]. Whole-genome sequencing (WGS) provides the most comprehensive view of genetic variation and is considered the gold standard for phylogenetic inference. Nevertheless, the widespread implementation of WGS for routine epidemiological surveillance remains challenging in many endemic countries due to its high costs, technical and analytical complexity, and the large volumes of data generated [20,21].

Previously, we demonstrated that selected highly variable genomic regions can accurately recapitulate the phylogeny obtained from complete coding sequences of DENV [22]. Herein we present the identification and validation of specific genomic regions from the four DENV serotypes, WNV, ZIKV, and YFV, useful for accurately reconstructing their complete phylogeny and facilitating the inference of lineage dispersion across geographic regions. This approach could provide a more accessible and practical alternative to whole-genome sequencing for epidemiological surveillance in resource-limited settings.

## 2. Materials and Methods

### 2.1. Genetic Variability Analysis

A total of 1115, 446, 2343, and 7844 complete genome sequences of ZIKV, YFV, WNV, and DENV, respectively, were downloaded from the BV-BRC database [23] between 16 August 2024, and 17 October 2024.

Sequences with available collection year and location information were selected. To ensure data quality, sequences containing three or more consecutive unassigned nucleotides or shorter than 9500 nt were excluded. Non-human-derived sequences were also removed. To mitigate potential long-branch attraction artifacts and reduce redundancy bias, highly divergent and 100% identical duplicate sequences were discarded [24]. In addition, sequences that appeared as outgroups in the phylogenetic tree were removed to further minimize long-branch attraction.

DENV serotypes were identified with the Genome Detective Typing Tool [25]. Sequences were aligned separately by serotype. Within each alignment, redundancy was further reduced to 99% identity using a mean-distance tree, yielding final datasets of 549 DENV-1, 513 DENV-2, 568 DENV-3 and 538 DENV-4 sequences, together with 486 ZIKV, 271 YFV and 1898 WNV sequences (hereafter “Sequence Set 1”). A comprehensive list of these sequences, including their accession numbers and associated metadata, is available in Supplementary File S2.

Alignments for each virus (and DENV serotype) were scanned for genetic variability by counting nucleotide changes in non-overlapping sliding windows of 300, 500, 700, 900, 1500, 2100 and 2700 nt relative to the consensus sequence (most frequent nucleotide at each position), non-overlapping windows were chosen to minimize redundancy in the analysis, ensuring that each region contributed unique and independent information, rather than repeatedly including the same nucleotide positions as could occur with overlapping windows. The specific window sizes were selected to cover a broad range of possibilities: smaller windows (300–900 nt) allow detection of variability in shorter genomic regions, while larger windows (1500–2700 nt) were defined as multiples of the smaller windows (e.g., 300 → 900, 500 → 1500, 700 → 2100, 900 → 2700 nt), facilitating concatenation of coding regions. This design enabled the identification of informative genomic regions suitable for concatenation and phylogenetic reconstruction. The specific genomic coordinates for all selected high- and low-variability regions for each virus are provided in Supplementary File S2.

### 2.2. Phylogenetic Analysis

Sequence redundancy within Sequence Set 1 was reduced using methods adapted to the initial dataset characteristics to produce representative subsets for each virus/serotype. For ZIKV and YFV, which exhibited lower initial redundancy, a “triplet reduction” approach was applied: sequences were first ordered by pairwise similarity using Jalview and subsequently grouped into non-overlapping triplets. Within each triplet, one representative sequence was retained, thereby eliminating redundancy while maintaining ~99% identity. For the more redundant WNV and DENV serotypes datasets, redundancy was reduced to 99% using a mean distance tree. Each resulting set comprised 180 sequences for each virus (and DENV serotype), which were randomly split into six replicate datasets of 30 sequences for downstream analyses. From these datasets, high- and low-variability regions were extracted at lengths of 300, 500, 700, 900, 1500, 2100, and 2700 nt. Additionally, concatenated datasets were constructed by joining the three high-variability regions (Con-Hi) and, separately, the three low-variability regions (Con-Low), resulting in total lengths of 900 (3 × 300 nt), 1500 (3 × 500 nt), 2100 (3 × 700 nt), and 2700 nt (3 × 900 nt).

Phylogenetic trees for the complete coding sequence (CSD) and for each individual and concatenated region were inferred under the GTR + G + I (General Time Reversible model with Gamma distribution and a proportion of invariant sites) substitution model, selected by ModelFinder using the Bayesian information criteria (BIC) [26]. Bayesian inference was performed using the MrBayes software v3.2.7 [27] employing a substitution model with six relative rates, a gamma distribution and a proportion of invariant sites for 50,000,000 states. The CSD tree of each replicate dataset served as a reference. Trees generated from the variable regions were compared to their respective reference tree using three metrics: mean posterior probability (PP) (as a measure of node support) [28], K-score (to quantify topological incongruence) [29], and Scale factor (to assess relative branch length differences) [30].

### 2.3. Phylogeographic Analysis

For phylogeographic inference, one representative sequence per country per year was selected from Sequence Set 1 and realigned by virus. Genotypes and lineages were assigned with the Genome Detective Typing Tool [31]. Sequences exhibiting anomalous temporal signals, identified by assessing root-to-tip divergence versus sampling date correlation were removed using TempEst v1.5.3 [32], specifically, sequences whose residuals deviated by three or more standard deviations from the regression line were excluded. The final datasets for phylogeographic analysis comprised 78 ZIKV, 67 YFV, 136 WNV, 177 DENV-1, 201 DENV-2, 140 DENV-3, and 98 DENV-4 sequences (the accession numbers and associated metadata for all sequences included in phylogeographic datasets are provided in Supplementary File S2). For WNV, in addition to conducting the analysis using all sequences together, a separate analysis was performed for each lineage, due to the substantial inter-lineage divergence, which made it difficult to estimate a common ancestor. For DENV-1 and DENV-3, the number of states exceeded 600 million and no ancestral reconstruction was obtained. Consequently, separate phylogeographic analyses were conducted using grouped subsets of sequences for these viruses.

Time-resolved phylogenetic trees were estimated using a Markov chain Monte Carlo (MCMC) approach implemented in BEAST v1.10.4 [33]. The most probable geographic location of the isolates was inferred through ancestral state reconstruction in BEAST, based on Bayesian inference along the phylogenetic tree. All analyses employed the GTR nucleotide substitution model [26], an uncorrelated relaxed clock model with log-normal distribution and a constant-size coalescent prior. MCMC chains were run for two billion iterations, with a 10% burn-in and sub sampling for every 100,000 iterations. Convergence and adequate mixing were confirmed by ensuring effective sample sizes (ESS) exceeded 200 for all relevant parameters using Tracer v1.7.2 [34]. To generate the maximum clade credibility (MCC) tree, TreeAnnotator v1.10.4 [35] was used with a 25% burn-in and posterior mean node heights. Finally, we visualized the phylogenetic trees in FigTree v1.4.4 [36].

## 3. Results

### 3.1. Genetic Variability Analysis

**Figure 1:**
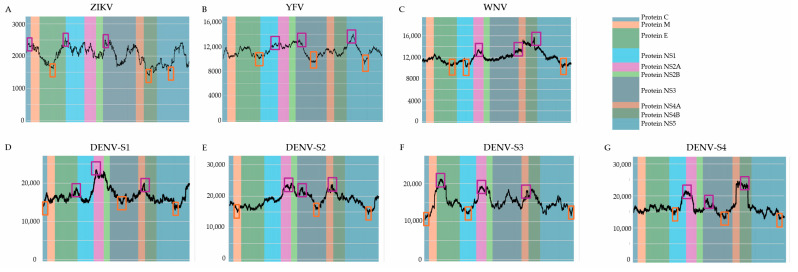
(**A**–**G**) Genetic variability analysis in ZIKV (**A**), YFV (**B**), WNV (**C**), DENV serotype 1 (**D**), DENV serotype 2 (**E**), DENV serotype 3 (**F**), and DENV serotype 4 (**G**) using a 700 nt sliding window. The background represents the position of the proteins relative to the reference genome. Y-axis: total number of changes per evaluated window. Peaks of high and low variability are highlighted.

The genome-wide genetic variability analysis conducted for ZIKV, YFV, WNV, and the four DENV serotypes revealed distinct patterns of high and low variability specific to each viral gene, although some common variable and stable regions were observed across all viruses (Figure 1A–G). For ZIKV, the highest number of nucleotide changes were detected in regions within the C, E and NS3 genes, whereas the lowest-variability regions were within the E, NS4B and NS5 genes (Figure 1A). For YFV, the most variable regions lay in NS1, NS3 and NS5, while the lowest-variability regions were in E, NS3 and NS5 (Figure 1B). WNV exhibited increased variability in NS2A, NS3 and NS4B, and lowest variability in E, NS1 and NS5 (Figure 1C). Among DENV serotypes, DENV-1 variability peaked in E, NS2A and NS4A, with C, NS3 and NS5 the least variable (Figure 1D); DENV-2 had the most changes in NS2A, NS3 and NS4A and the fewest in M, NS3 and NS5 (Figure 1E); DENV-3 variability was highest in E, NS2A and NS4B and lowest in C, NS1 and NS5 (Figure 1F); and DENV-4 variability peaked in NS2A, NS3 and NS4B (with NS1 also variable) while NS5 was least variable (Figure 1G). Despite these virus-specific patterns, some regions showed conserved variability across all flaviviruses: a high-variability region in NS3 (ZIKV, YFV, WNV, DENV-2, DENV-4), a high-variability NS2A region (WNV and all DENV serotypes), a low-variability region in NS5, and a low-variability region in E (ZIKV, YFV, WNV).

### 3.2. Phylogenetic Analysis

#### 3.2.1. ZIKV Phylogenetic Analysis

The ZIKV phylogeny inferred from the complete coding sequence (CDS) exhibited strong support (mean PP 0.96–0.98). Trees from highly variable regions consistently outperformed those from low-variability regions of the same length: mean PP remained low at 300 nt (max 0.39) but exceeded 0.93 at 2700 nt for the top region (Table S1; Figure S1A). Topological incongruence (K score) fell from 0.008–0.020 at 300 nt to 0.002–0.007 at 2700 nt (Table S2; Figure S1B), and branch-length accuracy (Scale Factor) converged on 1.0 with increasing length (Table S3; Figure S1C). The 2700 nt concatenated high-variability set yielded the most accurate reconstruction (mean PP 0.93, CI 0.90–0.96; K 0.002, CI 0.002–0.003; Scale 0.98, CI 0.95–1.01), approximating the CDS tree (mean PP 0.97; K 0; Scale 1), and was chosen for phylogeographic inference.

#### 3.2.2. YFV Phylogenetic Analysis

The YFV CDS reference tree exhibited high node support (mean PP: 0.95–0.98). Consistent with ZIKV results, tree support improved with increasing sequence length, with high-variability regions consistently outperforming low-variability regions. Mean PP values across all regions remain low at 300 nt (maximum 0.57), but at 2700 nt, mean PP exceeded 0.92 for the top-performing region (Table S1, Figure S2A). Topological incongruence (K-score) declined markedly with longer regions, from 0.11–0.29 at 300 nt to 0.01–0.08 at 2700 nt (Table S2, Figure S2B). Branch length accuracy (Scale Factor) also improved, narrowing from 1.45–4.13 at 300 nt to 0.91–1.80 at 2700 nt (Table S3, Figure S2C). Overall, the 2700 nt high-variability NS3 region provided the most robust performance (mean PP: 0.90; CI: 0.88–0.94; K-score: 0.031; CI: 0.025–0.037; Scale Factor: 1.03; CI: 0.98–1.09) compared to the CDS (mean PP: 0.98; CI: 0.96–0.99; K-score: 0; Scale Factor: 1) and was selected for phylogeographic inference.

#### 3.2.3. WNV Phylogenetic Analysis

The WNV CDS tree was well supported (mean PP 0.89–0.97). High-variability regions again outperformed low-variability ones: mean PP rose from ≤0.46 at 300 nt to >0.85 at 2700 nt (Table S1; Figure S3A), K scores fell from 0.14–0.36 to 0.03–0.28 (Table S2; Figure S3B), and Scale Factors improved from 1.48–2.62 to 1.04–1.86 (Table S3; Figure S3C). The 2700 nt NS3 region gave the most accurate reconstruction (mean PP 0.85, CI 0.80–0.90; K 0.078, CI 0.061–0.095; Scale 1.12, CI 1.09–1.15) and was chosen for phylogeographic inference.

#### 3.2.4. DENV Phylogenetic Analysis

For all four DENV serotypes, CDS trees had high node support (mean PP 0.89–0.99). Tee support improved substantially with increasing region length, mean PP values across all serotypes were low at 300 nt (maximum 0.70) but at 2700 nt, mean PP exceeded 0.97 for the top-performing regions across all serotypes (Table S1, Figure S4A–D). Topological incongruence (K-score) decreased progressively with sequence length, from 0.03–0.06 at 300 nt to 0.01–0.02 at 2700 nt (Table S2, Figure S5A–D). Branch length accuracy (Scale Factor) converged toward 1.0 as length increased, with variability narrowing from 0.41–0.64 (minimum) and 1.37–3.36 (maximum) at 300 nt to 0.77–0.89 (minimum) and 1.13–1.74 (maximum) at 2700 nt (Table S3, Figure S6A–D). Based on a combined assessment of all three metrics, the following 2700 nt regions were selected for phylogeographic inference: the high-variability NS4A region for DENV-1 (mean PP: 0.88; CI: 0.85–0.92; K-score: 0.019; CI: 0.016–0.023; Scale Factor: 1.01; CI: 0.98–1.03); concatenated high-variability regions for DENV-2 (mean PP: 0.94; CI: 0.91–0.98; K-score: 0.055; CI: 0.038–0.071; Scale Factor: 1.09; CI: 1.03–1.15); the high-variability E region for DENV-3 (mean PP: 0.90; CI: 0.82–0.97; K-score: 0.020; CI: 0.016–0.025; Scale Factor: 1.01; CI: 0.98–1.05); and the high-variability NS3 region for DENV-4 (mean PP: 0.90; CI: 0.87–0.96; K-score: 0.018; CI: 0.010–0.025; Scale Factor: 1.00; CI: 0.97–1.03), all closely approximating their respective CDS reference trees (mean PP: 0.96–0.98; K-score: 0; Scale Factor: 1).

### 3.3. Phylogeographic Analysis

#### 3.3.1. ZIKV Phylogeographic Analysis

**Figure 2:**
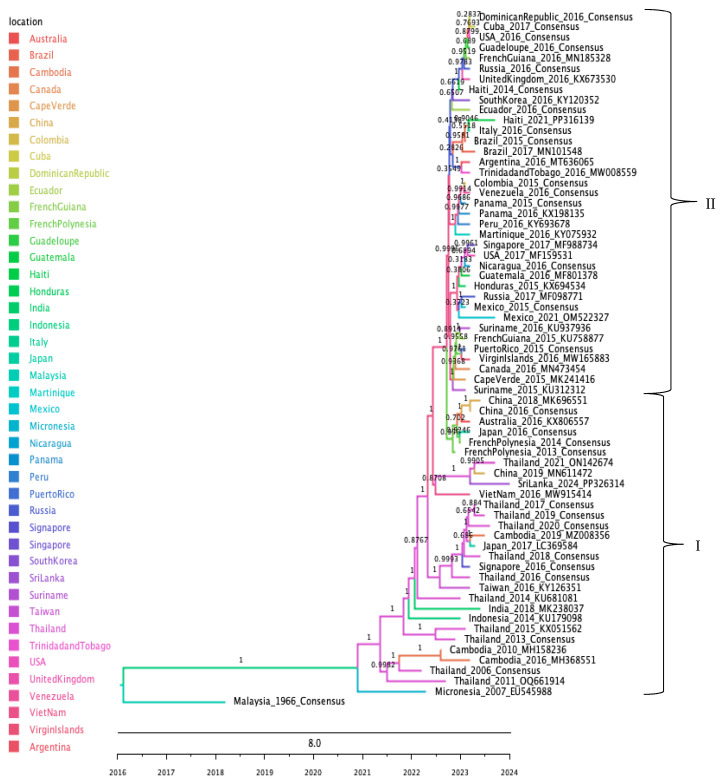
Maximum clade credibility phylogenetic tree of ZIKV, color-coded by geographic origin. All sequences belong to the Asian genotype, with two representative clades identified: Clade I (I) and Clade II (II).

Ancestral state reconstruction of the ZIKV Asian-genotype placed the most probable ancestral node in Indonesia (PP = 0.97) and revealed two well-supported clades, which we labeled Clade I and Clade II for ease of identification: Clade I, comprises early-diverging sequences mainly from Southeast Asia, with secondary introductions into Oceania; and Clade II contains predominantly American sequences sampled in 2014–2015, along with four European and two Asian isolates that likely represent imported cases (Figure 2).

#### 3.3.2. YFV Phylogeographic Analysis

**Figure 3:**
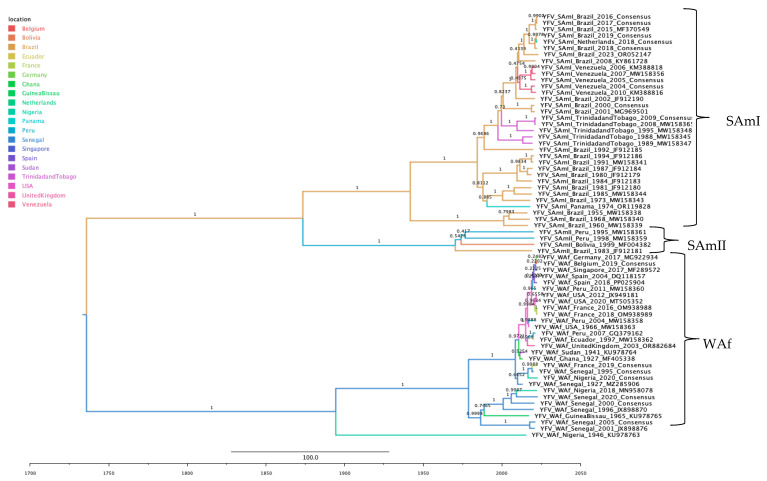
Maximum clade credibility phylogenetic tree of YFV, color-coded by geographic origin, with clades indicated for genotypes South America I (SAm I), South America II (SAm II), and West Africa (WAf).

Phylogeographic reconstruction for YFV revealed clustering by genotype and the corresponding geographical distribution of each sequence, as well as a temporally structured topology. The ancestral YFV node was inferred in Senegal, giving rise to the West Africa (WAf) genotype and a common ancestor for the South American genotypes (SAm I and SAm II). The node for the South American ancestor was in Brazil, which then diversified into the SAm I lineage (rooted in Brazil) and the SAm II lineage (rooted in Peru). Sequences from both clades were predominantly South American, although a sequence from Europe was identified in the SAm I clade, most likely representing an imported case. The WAf clade showed persistence of the ancestral lineage in Senegal, and a subsequent dissemination to Africa, Europe, and the Americas; a sequence detected in Asia is presumably an imported case. The analysis also resolved clear temporal structuring, distinguishing Brazilian sequences from the early 2000s from those collected after 2008, and separating other historic clades based on collection dates pre- and post-1970 (Figure 3).

#### 3.3.3. WNV Phylogeographic Analysis

**Figure 4:**
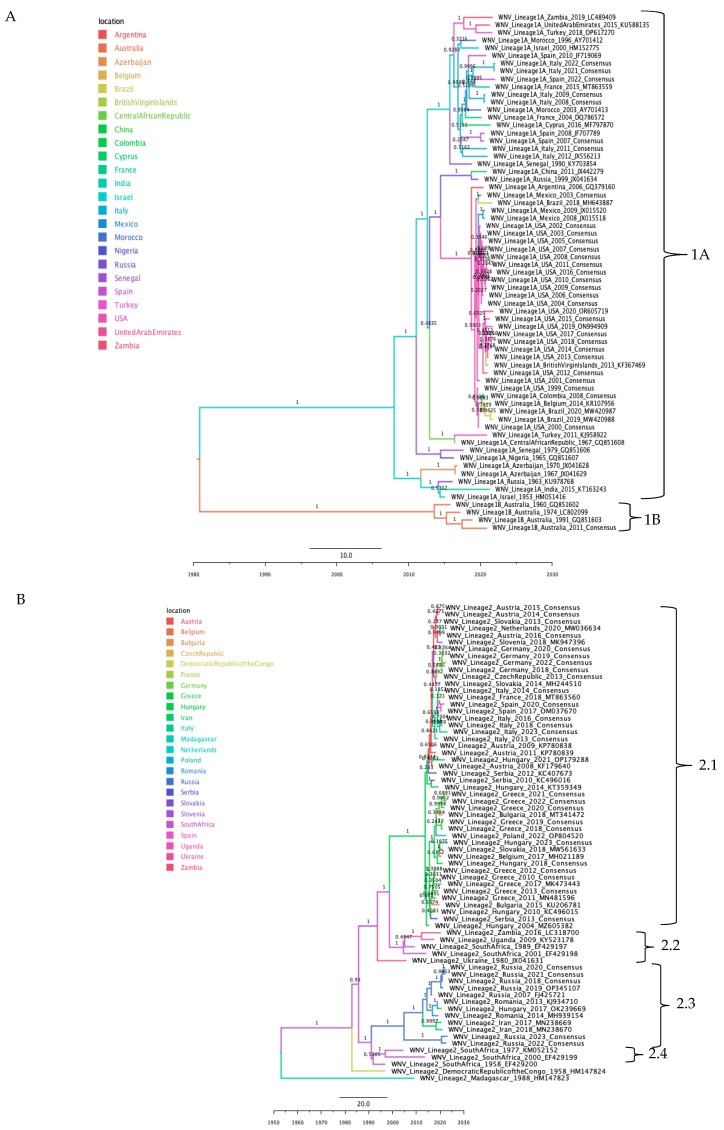
Maximum clade credibility phylogenetic tree of WNV lineages, color-coded by geographic origin. (**A**) Lineage 1, with clades 1A and 1B indicated. (**B**) Lineage 2, with clades labeled according to geographic distribution (2.1, 2.2, 2.3, and 2.4).

The phylogeographic reconstruction of WNV revealed high inter-lineage genetic distances that precluded fine-scale subclade analysis (Figure S7). Therefore, each lineage was analyzed separately (Figure 4A,B). For lineage 1, clades were organized by lineage, sub-lineage, and geographic origin. The ancestral node of Lineage 1 was traced to Australia. This lineage bifurcated into sub-lineage 1A, with its probable origin in Israel and broad intercontinental distribution across the Americas, Asia, Europe, and Africa. Sub-lineage 1B, remained rooted in Australia, the exclusive site of all recorded 1B sequences (Figure 4A).

For Lineage 2, the ancestral root was placed in Madagascar. A primary bifurcation separated the outgroup sequence from a major South African node, which gave rise to subclades designated as 2.1, 2.2, 2.3, and 2.4. From this South African node, one branch led to a subclade originating in the Democratic Republic of Congo. Another major branch, also rooted in South Africa, diversified further. This branch split into subclade 2.4 (centered in South Africa) and subclade 2.3 (rooted in Russia, with subsequent spread to Europe and Asia). A final split from the main South African branch led to subclade 2.2 (originating in South Africa and distributed across Africa) and subclade 2.1 (originating in Hungary and distributing across Europe) (Figure 4B).

#### 3.3.4. DENV-1 Phylogeographic Analysis

**Figure 5:**
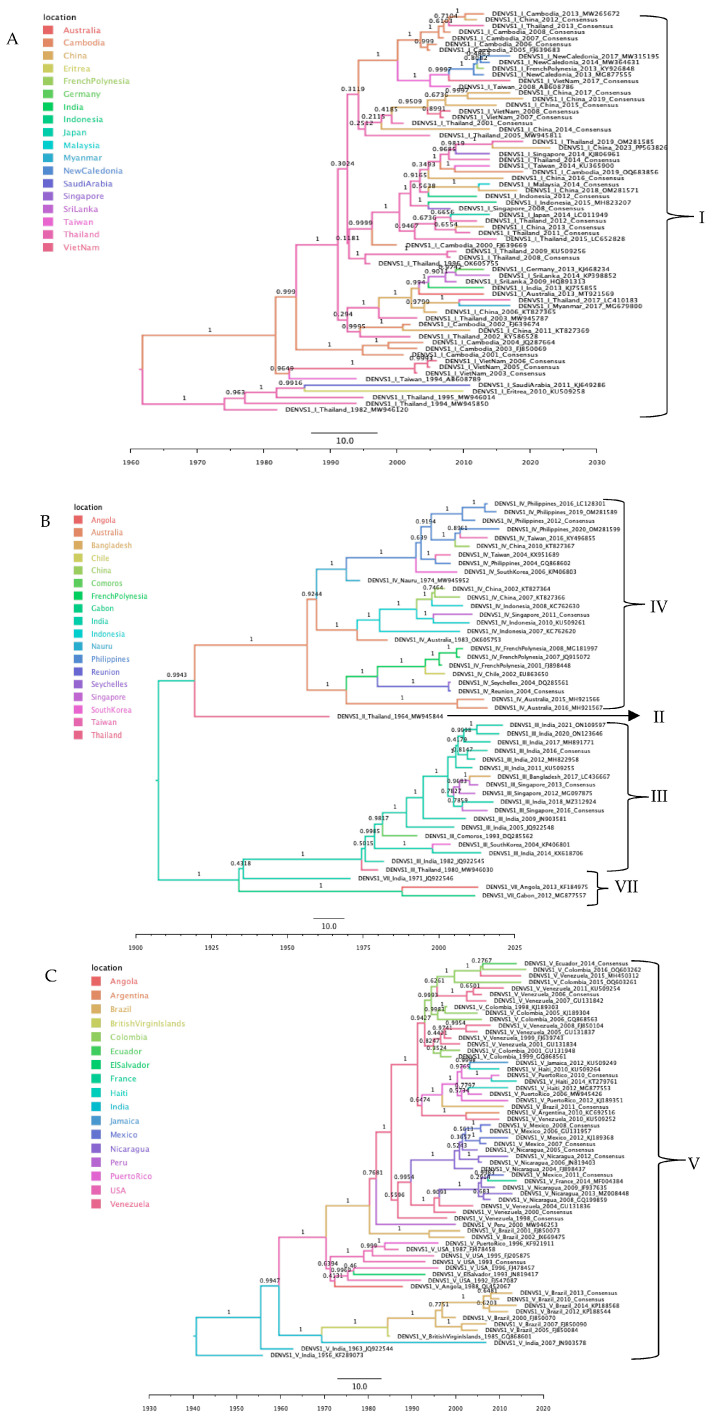
Maximum clade credibility phylogenetic tree of DENV-1. (**A**) Genotype I; color-coded by geographic origin. (**B**) Genotypes II, III, IV, and VII; color-coded by geographic origin with clades annotated by genotype. (**C**) Genotype V; color-coded by geographic origin.

The phylogeographic reconstruction of DENV-1 did not converge after 600 million MCMC states (Figure S8). Therefore, the dataset was divided into smaller subsets for phylogeographic inference (Figure 5A,B). In each subset, sequences clustered by genotype and geographic origin.

For the first subset (genotype I), the ancestral state reconstruction placed the ancestral node in Thailand. Sequences of this genotype were predominantly found in Asia but also appeared in Oceania, Africa, and Europe, the latter likely corresponding to an imported case (Figure 5A and Figure S8).

For the second subset (genotypes II, III, IV, and VII), the root was inferred in India, revealing a topology structured by both genotype and geographic origin. An initial bifurcation produced two main branches. The first branch split into genotype II (root in Thailand) and genotype IV (root in Indonesia), the latter found across Asia and Oceania, with isolated sequences detected in the Americas and Africa. The second branch divided into genotype VII (root in Gabon) and a lineage that further split into genotype III (root in India; predominantly Asian isolates) and an additional cluster of genotype VII sequences (probable origin in India) (Figure 5B and Figure S8).

For the third subset (genotype V), the phylogeographic analysis placed the root in India. Genotype V circulates throughout Asia and the Americas, with additional sequences detected in Africa and Europe, likely representing imported cases (Figure 5C and Figure S8).

#### 3.3.5. DENV-2 Phylogeographic Analysis

The phylogeographic analysis of DENV-2 revealed that clades cluster consistently by genotype and geographic origin and exhibit temporal structuring. The root was placed in Cambodia, from which an initial bifurcation emerged. One branch gave rise to the American genotype, whose most probable node location was Puerto Rico. Subsequent sequences are mainly from the Americas, except for two from Asia and one from Oceania. The other branch split into two lineages. The first corresponds to the Cosmopolitan genotype, rooted in Singapore. Although this genotype was predominantly identified in Asia, isolates were also recorded in Oceania, Africa, and a solitary case in Europe (presumably imported). The second lineage split into the Asian American genotype, rooted in Vietnam; this clade is organized into two well-defined clusters: one comprising sequences from Asia and the other from the Americas. The final subdivision gave rise to the Asian I and Asian II genotypes. The ancestral node of the Asian II genotype was located in Colombia including sequences from the Americas, Oceania, and Asia, whereas the Asian I genotype is rooted in Thailand, with sequences primarily from Asia.

**Figure 6:**
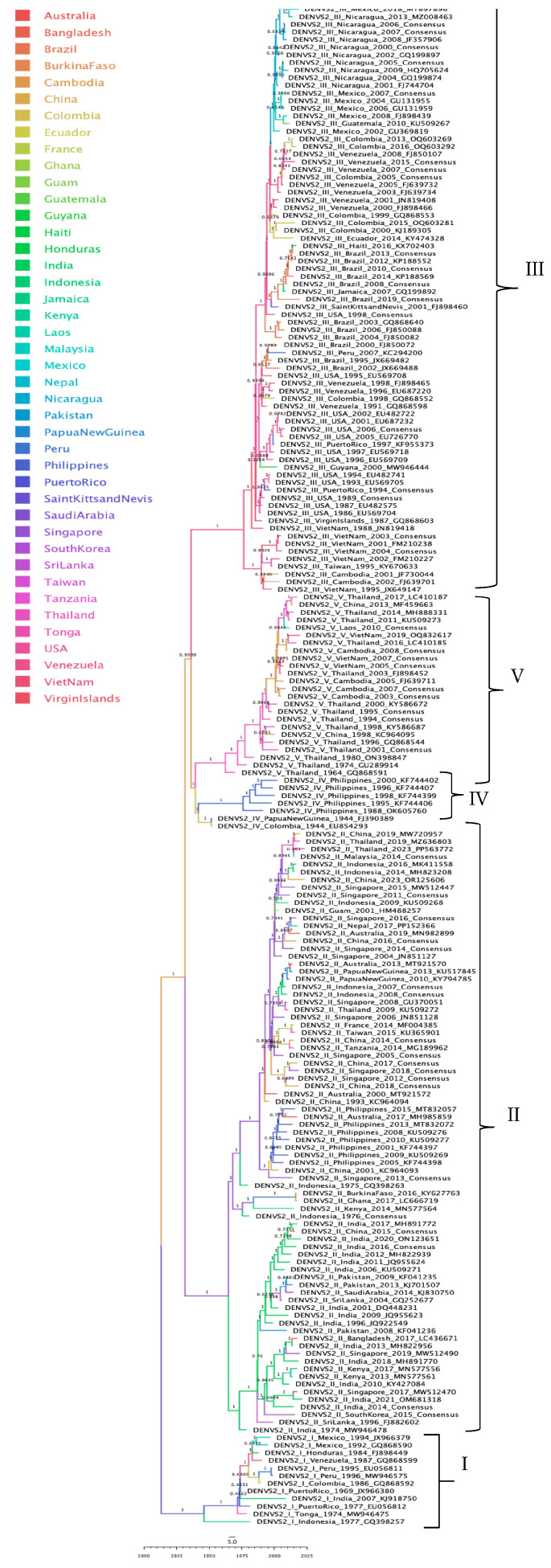
Maximum clade credibility phylogenetic tree of DENV-2, color-coded by geographic origin, with clades indicated for genotypes I (American), II (Cosmopolitan), III (Southern Asian–American), IV (Asian II), and V (Asian I).

Temporal structures were evident in genotype III, Venezuelan sequences were separated into groups corresponding to sequences from 2000 to 2005 and those from 2007, 2008, and 2015. Brazilian isolates exhibited a temporal split between those from early 2000 and samples collected post-2008. United States sequences also displayed divergence between those from late 1990 and those from early 2000. Finally, in genotype V, Thai sequences were grouped according to well-defined temporal periods of 1960s, 1970s, 1990s, and early 2000s (Figure 6).

#### 3.3.6. DENV-3 Phylogeographic Analysis

**Figure 7:**
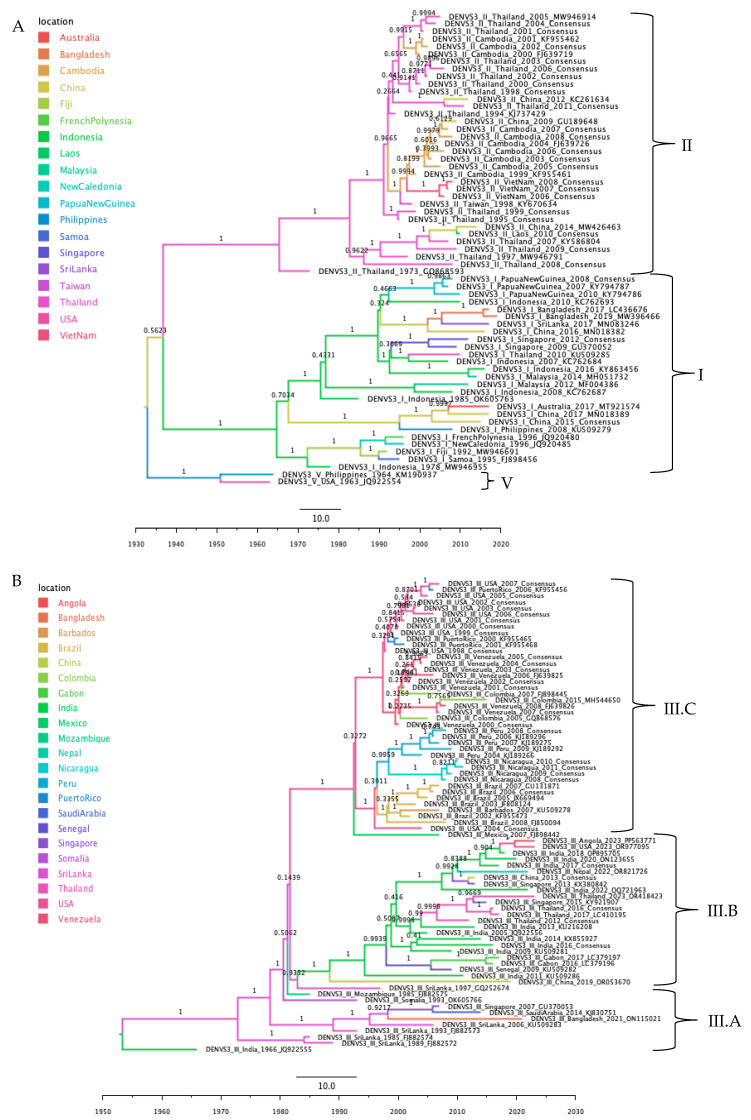
Maximum clade credibility phylogenetic tree of DENV-3. (**A**) Genotypes I, II, and V, color-coded by geographic origin with clades labeled by genotype. (**B**) Genotype III, color-coded by geographic origin with clades labeled by lineages III.A, III.B, and III.C.

The phylogeographic reconstruction of DENV-3 failed to converge after 600 million MCMC states (Figure S9). Therefore, the dataset was divided into smaller subsets for phylogeographic inference (Figure 7A,B). In these subsets sequences clustered by genotype, lineage and geographic origin. The first subset comprised sequences of genotypes I, II, and V. The most likely origin of this group was China, were the root node initially bifurcated. One branch gave rise to genotype V, rooted in Philippines and including one USA and one Philippine sequence. The other branch split into two lineages: genotype I (rooted in Indonesia and distributed across Asia and Oceania) and genotype II (rooted in Thailand and predominantly distributed in Asia) (Figure 7A and Figure S9).

The second subset comprised sequences from genotype III. This genotype subsequently diverged into three lineages: A, B, and C. Clade III.C consists primarily of sequences from the Americas, with one African isolate, whereas clades III.A and III.B include sequences from Asia and Africa; clade III.B, also contains a single sequence from the Americas, presumably imported (Figure 7B and Figure S9).

#### 3.3.7. DENV-4 Phylogeographic Analysis

**Figure 8:**
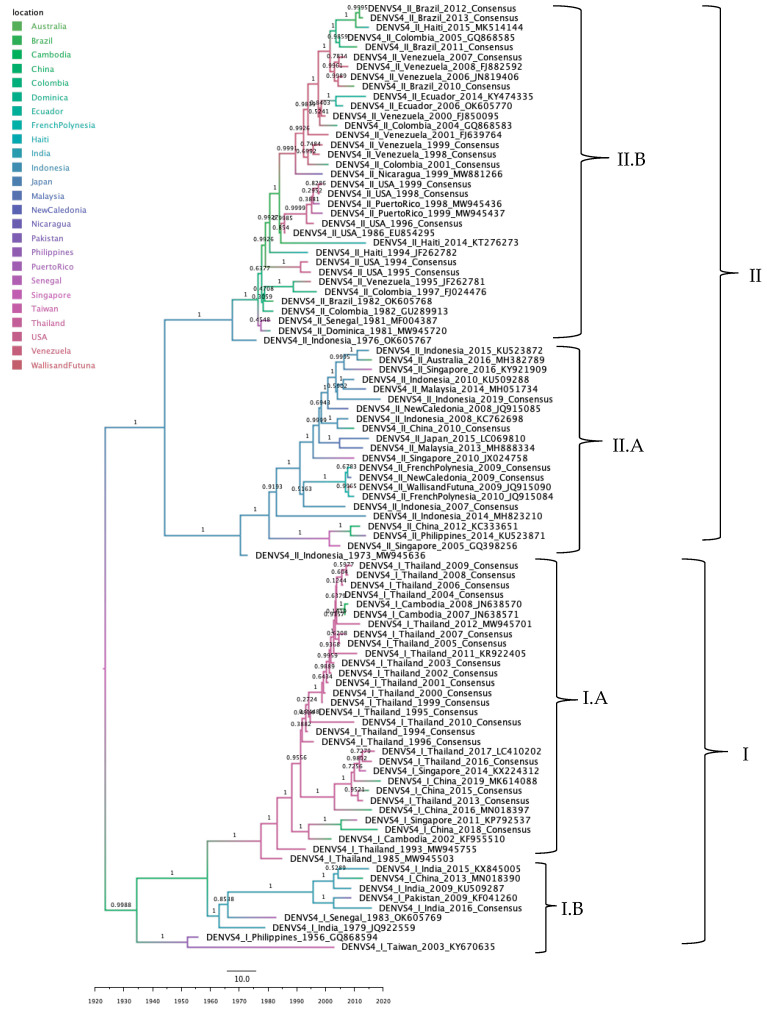
Maximum clade credibility phylogenetic tree of DENV-4, color-coded by geographic origin, with clades indicated for genotypes I and II, each subdivided into lineages I.A, I.B, II.A, and II.B.

The ancestral-state reconstruction of DENV-4 indicated that clade clustering was determined by genotype, lineage, and geographic origin. The ancestral node of DENV-4 was placed in Taiwan, from which a bifurcation gave rise to genotypes I and II. Genotype II was rooted in Indonesia, where it further split into lineages II.A and II.B. Lineage: Lineage II.A comprises sequences from Oceania and Asia, whereas lineage II.B includes predominantly American sequences, with one African and one Asian isolate. In contrast, genotype I was rooted in China, and bifurcated into lineages I.A and I.B, whose descendant sequences are distributed across Asia (Figure 8).

## 4. Discussion

This study identified genomic regions that capture the functional diversity of four DENV serotypes, WNV, ZIKV and YFV, and that reliably recapitulate full-genome phylogenies for surveillance purposes. Genetic variability analysis showed that, despite belonging to different species, these viruses share regions of high and low variability. ZIKV, YFV, and WNV exhibit a highly variable region in the NS3 protein; all four DENV serotypes share a similar region in NS2A; and all analyzed viruses present a conserved, low-variability region in NS5. This protein contains both a methyltransferase (MTase) and an RNA-dependent RNA polymerase (RdRp) domain, which are essential for RNA capping, methylation, and replication. Due to its critical functions, NS5 is highly conserved at structural and sequence levels [37,38]. NS2A has been implicated in IFN-β inhibition, suggesting that its high variability may be linked to immune evasion mechanisms [39]. Similarly, while NS3 is indispensable for viral replication, it has been implicated in evasion of the host innate immune response through interference with signaling proteins involved in antiviral defense. The high variability observed in NS3 across flaviviruses may contribute to differential interactions with host immune factors that, potentially facilitate immune escape [40,41,42].

To evaluate the capacity of the identified variable regions to recapitulate the phylogeny inferred from the complete coding sequence (CDS), we compared phylogenetic trees using three key metrics. First, tree support, measured as the mean (PP), quantifies statistical confidence in the observed clades; values close to 1 indicate strong topological support [28]. Second, topological incongruence, evaluated with the K-score, assesses structural similarity to the CDS reference tree; a low K-score (ideally close to 0) denotes high fidelity in reproducing the branching pattern [29]. Third, relative branch-length accuracy, determined by the Scale Factor derived from the branch length distance (BLD), evaluates how well the branch lengths (representing genetic change) in the variable-region tree reflect those of the CDS tree [30]. A Scale Factor of 1 indicates perfect agreement; values > 1 suggest shorter branches (underestimation of change), and values < 1 indicate longer branches (overestimation of change) in the test tree.

Our findings indicate that longer genomic regions yielded values that more closely approximate the CDS phylogeny. Likewise, highly variable regions demonstrated a greater capacity to robustly reproduce the CDS phylogeny. These findings are consistent with a previous study showing that the scarce phylogenetic signal contained in low-variable sequences poses a more significant issue than the potential noise introduced by highly divergent regions [19].

Several studies have employed specific genomic regions for phylogeographic inference in viruses, including the Influenza (family Orthomyxoviridae), Chikungunya (family Togaviridae) and members of the Flaviviridae family, such as WNV, DENV, and Kyasanur Forest disease virus. However, these analyses were restricted to single outbreaks, with isolates from the same spatial and temporal context and insufficient viral diversity to discriminate between lineages [9,43,44,45,46]. Moreover, not all genomic regions are suitable for phylogeographic inference. For example, while whole-genome phylogenies successfully resolved the East-Central-South-African (ECSA) lineages of chikungunya, analyses based solely on the E1 gene did not [43]. A further limitation of whole-genome-based studies is the reduced number of genomes that can be obtained, which often limits phylogeographic resolution. When only a limited number of genotypes or lineages are included, the phylogenetic signal obtained is weak, resulting in incongruent and poorly supported evolutionary hypotheses [47,48,49].

Our phylogeographic analyses demonstrated that regions of 2700 nt were adequate and sufficient to reliably resolve the various genotypes or lineages of all viruses included in this study, as well as to trace their geographic distribution with high resolution. This type of analysis is essential for understanding current patterns of viral spread, especially in a global context; especially as mosquito vectors expand into new ecological niches and international travel accelerates reemergence and dissemination [50]. In this context, our analysis allowed for the identification of probable imported cases for all studied viruses, except for DENV-4. This may be due to the limited number of sequences analyzed, as DENV-4 infections are often asymptomatic or mild, leading to fewer detected cases and consequently a lower number of sequences available for epidemiological and genomic studies [51,52,53].

ZIKV is classified into African and Asian lineages; the latter is associated with recent epidemics and severe disease, it is more efficient at establishing infection in the mosquito midgut, leading to a higher transmission potential compared to the African lineage [54,55,56]. This may explain why all sequences in our dataset belong to the Asian lineage. Our variability analysis supports this observation, showing relatively limited genetic changes are compared to those found in the other viruses included studied. The reconstructed spread patterns, including the Asian genotype origin of ZIKV and the Brazilian outbreak cluster of YFV, are consistent with established literature [54,55,56,57,58,59]. In addition, one Canadian sequence clustered within its expected geographic clade, which we interpreted as a probable imported case since local transmission of this virus has not been documented in this country [60]. This finding validates the resolution of our approach.

For YFV, a major outbreak was reported in Brazil between 2016 and 2019. This event is reflected in our phylogenetic tree, where the outbreak sequences form a well-defined cluster [57,58,59].

In WNV, we observed substantial genetic divergence (>6%) between the analyzed lineages, and our variability analysis identified a correspondingly high frequency of nucleotide changes. Such divergence levels suggest that these lineages may represent distinct genotypes rather than merely divergent lineages. A proposition consistent with DENV genotype demarcation based on ~6% divergence [61]. Supporting this interpretation, phylogeographic analyses for each lineage separately, resulted in well-defined clades and exhibited unique geographic distributions. The classification of WNV lineages is not globally standardized and merits further discussion to establish more objective and reproducible criteria in future studies [62].

In the case of DENV-1, genotype VII was recently described [63]; however, in our phylogenetic tree, the sequences assigned to this genotype did not form a monophyletic group. This suggests that there is limited supporting evidence for its recognition as a distinct genotype, and more extensive studies are needed to robustly establish its classification.

It has been documented that the DENV-2 American genotype was displaced by the Asian–American genotype during the 1990s [64], which is supported by our phylogenetic tree. Sequences of the American genotype derive primarily from samples collected in the 1990s or earlier. In contrast, Asian American genotype sequences co-circulated with the American genotype but were mostly sampled after that decade and have since become predominant.

A previous study documented that DENV-3 genotype III lineage A was distributed in Asia, lineage B in both the Americas and Asia, and lineage C in the Americas [65]. Our results confirm an American distribution of lineage C. However, for lineages A and B, we observed discrepancies: our analysis indicated that lineage B is distributed across Asia and Africa, similarly to lineage A; nevertheless, lineage A showed higher prevalence in Asia, as only two sequences corresponded to African isolates. Notably, phylogeographic analyses for DENV-1 and DENV-3 faced convergence challenges, likely due to the large dataset sizes and potentially high divergence among sequences. Although model misspecification could be considered, the substitution model was selected using ModelTest and applied consistently across all viruses. This limitation primarily affects inter-genotype comparisons, while intra-genotype analyses remain robust and reliable.

For DENV-4, previous studies reported that genotype I circulates exclusively in Southeast Asia, whereas genotype II circulates in both Southeast Asia and the Americas [66]. In our analysis, sequences of genotype I formed a clade composed solely of Asian isolates. In contrast, genotype II split into two well defined clades, confirming the previously described geographic distribution, one clade being exclusive to the Americas and the other to Asia. In both genotypes, sequence clustering faithfully reflects lineage structure and its strong association with geographic origin.

In recent years, viral reintroductions have been documented as a phenomenon driven by multiple and complex factors that poses a significant public health risk [67,68]. Our analyses enabled inference of some of these events by examining the temporal patterns of viral sequences. In certain instances, sequences form clear and separate clusters within the phylogenetic tree, defined by both their collection date and their geographic location, which strongly suggests reintroductions. This pattern was particularly evident for YFV and DENV-2.

This study demonstrated that highly variable genomic regions can reliably recapitulate whole-genome phylogenies and offer valuable insights for the phylogeographic analysis of flaviviruses. These findings highlighted the importance of strategic region selection in genomic surveillance, particularly in contexts where whole-genome sequencing may not be feasible. A limitation of this study is the analyses restricted to sequences available in public databases that also met the required metadata criteria, which may have reduced the representation of certain geographic regions or time periods. Even so, by enabling more efficient approaches to monitor the evolution and spread of medically important flaviviruses, this approach may enhance precision and broaden the scope of molecular epidemiological surveillance. In addition, the regions identified here could be used as templates for the design of oligonucleotides targeting multiple flaviviruses to facilitate amplification strategies that can be coupled with portable sequencing devices. Recent studies have shown that targeted amplification of flavivirus genomic regions can be successfully integrated with portable sequencing devices, enabling real-time pathogen detection and identification even in resource-limited settings [69]. This suggests that the regions identified here could be directly adapted to portable sequencing workflows, thus reinforcing their practicality for field-based genomic surveillance.

## Data Availability

The sequence data used in this study were obtained from publicly available databases, primarily GenBank and the Bacterial and Viral Bioinformatics Resource Center (BV-BRC). A comprehensive list of all sequences analyzed, including their respective accession numbers, is provided in the Supplementary Materials.

## Supplementary Materials

The following supporting information can be downloaded at: https://www.mdpi.com/article/10.3390/tropicalmed10100277/s1, Table S1: Mean PP values for ZIKV, YFV, WNV and DENV serotypes; Table S2: Mean K-score values for ZIKV, YFV, WNV and DENV serotypes; Table S3: Mean Scale Factor values for ZIKV, YFV, WNV and DENV serotypes; Figure S1: Phylogenetic analysis of ZIKV; Figure S2: Phylogenetic analysis of YFV; Figure S3: Phylogenetic analysis of WNV; Figure S4: Tree Support Analysis for DENV Serotypes; Figure S5: Topological Incongruence Analysis for DENV Serotypes; Figure S6: Branch Length Analysis for DENV Serotypes; Figure S7: Phylogenetic tree of WNV; Figure S8: Phylogenetic tree of DENV-1; Figure S9: Phylogenetic tree of DENV-3.
