# Supplementary material for: Inferring Geographic Spread of Flaviviruses Through Analysis of Hypervariable Genomic Regions
Source: Trop Med Infect Dis. 2025 Sep 24;10(10):277. doi: 10.3390/tropicalmed10100277 (PMC12567808; doi:10.3390/tropicalmed10100277)
Supplement: Supplementary file 1 [file tropicalmed-10-00277-s001.zip › Suplemmentary Figures and Tables.pdf]

Supplementary Materials

Table S1. Mean PP values for ZIKV, YFV, WNV and DENV serotypes. The table shows the 95% confidence intervals of the mean for the analyzed regions across all evaluated segment lengths.

Virus	Region	Mean (CI 95%)						
		300 nt length	500 nt length	700 nt length	900 nt length	1500 nt length	2100 nt length	2700 nt length
ZIKV	C_Hi	0.30 (0.25 - 0.35)	0.41 (0.38 - 0.45)	0.42 (0.38 - 0.46)	0.54 (0.50 - 0.58)	0.65 (0.59 - 0.72)	0.69 (0.63 - 0.76)	0.79 (0.74 - 0.83)
	E_Hi	0.39 (0.36 - 0.42)	0.52 (0.44 - 0.61)	0.52 (0.44 - 0.60)	0.66 (0.58 - 0.75)	0.77 (0.70 - 0.84)	0.77 (0.69 - 0.86)	0.83 (0.78 - 0.89)
	NS3_Hi	0.27 (0.20 - 0.33)	0.42 (0.34 - 0.50)	0.48 (0.38 - 0.58)	0.51 (0.43 - 0.60)	0.62 (0.54 - 0.71)	0.73 (0.67 - 0.79)	0.81 (0.75 - 0.87)
	E_Low	0.12 (0.07 - 0.18)	0.22 (0.18 - 0.27)	0.34 (0.28 - 0.41)	0.40 (0.33 - 0.47)	0.71 (0.65 - 0.78)	0.78 (0.72 - 0.84)	0.81 (0.75 - 0.88)
	NS4B_Low	0.13 (0.10 - 0.17)	0.14 (0.08 - 0.20)	0.28 (0.23 - 0.33)	0.31 (0.26 - 0.37)	0.46 (0.40 - 0.52)	0.69 (0.62 - 0.76)	0.74 (0.69 - 0.79)
	NS5_Low	0.13 (0.10 - 0.17)	0.20 (0.18 - 0.22)	0.23 (0.20 - 0.26)	0.36 (0.31 - 0.42)	0.63 (0.60 - 0.67)	0.71 (0.68 - 0.74)	0.72 (0.70 - 0.74)
	Con_Hi				0.60 (0.53 - 0.67)	0.73 (0.68 - 0.77)	0.77 (0.71 - 0.82)	0.93 (0.90 - 0.96)
	Con_Low				0.32 (0.25 - 0.38)	0.43 (0.36 - 0.51)	0.57 (0.50 - 0.64)	0.92 (0.90 - 0.94)
YFV	NS1_Hi	0.51 (0.47 - 0.55)	0.60 (0.57 - 0.64)	0.67 (0.64 - 0.69)	0.70 (0.68 - 0.72)	0.70 (0.68 - 0.73)	0.84 (0.81 - 0.87)	0.86 (0.84 - 0.89)
	NS3_Hi	0.57 (0.54 - 0.61)	0.67 (0.65 - 0.70)	0.81 (0.79 - 0.84)	0.82 (0.80 - 0.85)	0.83 (0.78 - 0.87)	0.89 (0.86 - 0.92)	0.91 (0.88 - 0.94)
	NS5_Hi	0.49 (0.46 - 0.53)	0.58 (0.55 - 0.61)	0.64 (0.58 - 0.71)	0.73 (0.68 - 0.78)	0.75 (0.70 - 0.80)	0.77 (0.73 - 0.80)	0.77 (0.73 - 0.81)
	E_Low	0.41 (0.35 - 0.46)	0.46 (0.44 - 0.47)	0.55 (0.52 - 0.59)	0.60 (0.56 - 0.64)	0.65 (0.61 - 0.69)	0.72 (0.68 - 0.76)	0.77 (0.74 - 0.81)
	NS3_Low	0.40 (0.32 - 0.47)	0.54 (0.50 - 0.57)	0.60 (0.55 - 0.65)	0.64 (0.60 - 0.68)	0.82 (0.76 - 0.88)	0.88 (0.84 - 0.92)	0.89 (0.84 - 0.93)
	NS5_Low	0.38 (0.35 - 0.41)	0.47 (0.43 - 0.50)	0.50 (0.46 - 0.53)	0.57 (0.52 - 0.63)	0.72 (0.69 - 0.76)	0.73 (0.69 - 0.77)	0.73 (0.69 - 0.77)
	Con_Hi				0.70 (0.64 - 0.75)	0.76 (0.74 - 0.79)	0.88 (0.84 - 0.91)	0.90 (0.88 - 0.92)
	Con_Low				0.66 (0.62 - 0.71)	0.69 (0.64 - 0.75)	0.74 (0.69 - 0.80)	0.79 (0.73 - 0.85)
WNV	NS2A_Hi	0.32 (0.25 - 0.40)	0.47 (0.39 - 0.55)	0.48 (0.40 - 0.55)	0.52 (0.46 - 0.58)	0.61 (0.51 - 0.72)	0.66 (0.56 - 0.76)	0.73 (0.70 - 0.77)
	NS3_Hi	0.36 (0.32 - 0.39)	0.57 (0.50 - 0.63)	0.58 (0.51 - 0.65)	0.61 (0.55 - 0.66)	0.77 (0.71 - 0.82)	0.80 (0.73 - 0.87)	0.85 (0.80 - 0.90)
	NS4B_Hi	0.46 (0.37 - 0.54)	0.54 (0.47 - 0.61)	0.58 (0.52 - 0.64)	0.61 (0.56 - 0.65)	0.71 (0.66 - 0.75)	0.78 (0.74 - 0.81)	0.77 (0.71 - 0.82)
	E_Low	0.27 (0.20 - 0.34)	0.33 (0.26 - 0.40)	0.37 (0.33 - 0.42)	0.45 (0.42 - 0.49)	0.58 (0.49 - 0.66)	0.68 (0.63 - 0.72)	0.74 (0.67 - 0.81)
	NS1_Low	0.26 (0.20 - 0.32)	0.36 (0.29 - 0.42)	0.44 (0.33 - 0.55)	0.53 (0.43 - 0.63)	0.62 (0.55 - 0.70)	0.66 (0.59 - 0.73)	0.72 (0.64 - 0.81)
	NS5_Low	0.27 (0.23 - 0.31)	0.37 (0.34 - 0.40)	0.48 (0.37 - 0.58)	0.52 (0.40 - 0.63)	0.57 (0.47 - 0.67)	0.57 (0.46 - 0.68)	0.57 (0.47 - 0.68)
	Con_Hi				0.67 (0.61 - 0.73)	0.77 (0.71 - 0.83)	0.80 (0.76 - 0.85)	0.81 (0.76 - 0.86)
	Con_Low				0.38 (0.31 - 0.44)	0.48 (0.39 - 0.57)	0.60 (0.51 - 0.68)	0.71 (0.63 - 0.78)
DENV-1	E_Hi	0.60 (0.53 - 0.66)	0.68 (0.61 - 0.74)	0.78 (0.75 - 0.82)	0.80 (0.75 - 0.85)	0.85 (0.81 - 0.89)	0.87 (0.84 - 0.89)	0.90 (0.88 - 0.92)
	NS2A_Hi	0.53 (0.48 - 0.57)	0.65 (0.62 - 0.69)	0.69 (0.66 - 0.73)	0.77 (0.72 - 0.81)	0.84 (0.81 - 0.88)	0.86 (0.82 - 0.89)	0.88 (0.83 - 0.92)
	NS4A_Hi	0.51 (0.46 - 0.57)	0.62 (0.57 - 0.67)	0.65 (0.63 - 0.68)	0.75 (0.72 - 0.79)	0.83 (0.79 - 0.88)	0.86 (0.83 - 0.89)	0.88 (0.85 - 0.92)
	C_Low	0.34 (0.30 - 0.39)	0.55 (0.51 - 0.58)	0.67 (0.63 - 0.71)	0.70 (0.65 - 0.74)	0.81 (0.76 - 0.87)	0.82 (0.78 - 0.87)	0.87 (0.85 - 0.89)
	N3_Low	0.47 (0.41 - 0.53)	0.63 (0.58 - 0.67)	0.70 (0.66 - 0.74)	0.77 (0.71 - 0.83)	0.83 (0.80 - 0.87)	0.87 (0.81 - 0.93)	0.87 (0.84 - 0.91)
	NS5_Low	0.43 (0.37 - 0.49)	0.58 (0.55 - 0.61)	0.72 (0.69 - 0.75)	0.76 (0.72 - 0.81)	0.82 (0.79 - 0.86)	0.83 (0.79 - 0.87)	0.83 (0.79 - 0.87)
	Con_Hi				0.75 (0.71 - 0.80)	0.81 (0.77 - 0.85)	0.85 (0.83 - 0.87)	0.93 (0.90 - 0.95)
	Con_Low				0.69 (0.65 - 0.72)	0.78 (0.73 - 0.83)	0.80 (0.77 - 0.84)	0.84 (0.79 - 0.89)
DENV-2	NS2A_Hi	0.58 (0.52 - 0.65)	0.64 (0.56 - 0.72)	0.74 (0.70 - 0.79)	0.83 (0.77 - 0.88)	0.81 (0.76 - 0.87)	0.85 (0.81 - 0.90)	0.88 (0.84 - 0.92)
	NS3_Hi	0.60 (0.55 - 0.66)	0.73 (0.66 - 0.80)	0.79 (0.72 - 0.86)	0.82 (0.77 - 0.87)	0.90 (0.86 - 0.93)	0.90 (0.87 - 0.94)	0.88 (0.82 - 0.94)
	NS4A_Hi	0.64 (0.56 - 0.71)	0.74 (0.69 - 0.79)	0.79 (0.73 - 0.85)	0.82 (0.77 - 0.87)	0.92 (0.89 - 0.95)	0.92 (0.90 - 0.95)	0.94 (0.90 - 0.98)
	M_Low	0.57 (0.52 - 0.62)	0.67 (0.60 - 0.73)	0.78 (0.77 - 0.79)	0.81 (0.78 - 0.84)	0.85 (0.82 - 0.88)	0.84 (0.82 - 0.86)	0.89 (0.84 - 0.93)
	NS3_Low	0.64 (0.57 - 0.71)	0.76 (0.70 - 0.81)	0.79 (0.77 - 0.82)	0.84 (0.80 - 0.87)	0.86 (0.82 - 0.91)	0.86 (0.81 - 0.92)	0.92 (0.89 - 0.95)
	NS5_Low	0.47 (0.41 - 0.53)	0.61 (0.52 - 0.69)	0.70 (0.66 - 0.73)	0.80 (0.77 - 0.84)	0.87 (0.81 - 0.93)	0.88 (0.82 - 0.94)	0.87 (0.81 - 0.93)
	Con_Hi				0.82 (0.77 - 0.87)	0.86 (0.83 - 0.88)	0.92 (0.89 - 0.94)	0.94 (0.91 - 0.98)
	Con_Low				0.75 (0.71 - 0.79)	0.85 (0.80 - 0.91)	0.88 (0.82 - 0.94)	0.89 (0.84 - 0.93)
DENV-3	E_Hi	0.51 (0.45 - 0.57)	0.75 (0.70 - 0.80)	0.80 (0.75 - 0.86)	0.83 (0.76 - 0.89)	0.89 (0.82 - 0.95)	0.88 (0.81 - 0.95)	0.90 (0.82 - 0.97)
	NS2A_Hi	0.54 (0.47 - 0.61)	0.63 (0.57 - 0.70)	0.69 (0.60 - 0.78)	0.72 (0.65 - 0.80)	0.78 (0.72 - 0.84)	0.83 (0.78 - 0.87)	0.85 (0.80 - 0.90)
	NS4B_Hi	0.59 (0.53 - 0.64)	0.63 (0.56 - 0.70)	0.72 (0.66 - 0.79)	0.75 (0.67 - 0.83)	0.79 (0.73 - 0.85)	0.85 (0.81 - 0.89)	0.87 (0.83 - 0.92)
	C_Low	0.35 (0.31 - 0.38)	0.46 (0.39 - 0.53)	0.64 (0.55 - 0.72)	0.69 (0.62 - 0.77)	0.82 (0.79 - 0.86)	0.89 (0.84 - 0.94)	0.90 (0.83 - 0.96)
	NS1_Low	0.39 (0.34 - 0.44)	0.49 (0.41 - 0.58)	0.62 (0.56 - 0.68)	0.68 (0.62 - 0.73)	0.75 (0.68 - 0.81)	0.83 (0.77 - 0.89)	0.83 (0.78 - 0.89)
	NS5_Low	0.42 (0.32 - 0.52)	0.59 (0.51 - 0.66)	0.68 (0.62 - 0.75)	0.69 (0.62 - 0.77)	0.69 (0.61 - 0.77)	0.69 (0.62 - 0.77)	0.69 (0.62 - 0.77)
	Con_Hi				0.79 (0.76 - 0.82)	0.86 (0.84 - 0.89)	0.90 (0.87 - 0.94)	0.91 (0.89 - 0.94)
	Con_Low				0.70 (0.62 - 0.77)	0.76 (0.69 - 0.84)	0.83 (0.77 - 0.88)	0.84 (0.78 - 0.90)
DENV-4	NS2A_Hi	0.68 (0.64 - 0.72)	0.76 (0.72 - 0.81)	0.79 (0.74 - 0.84)	0.82 (0.76 - 0.89)	0.88 (0.83 - 0.93)	0.92 (0.89 - 0.95)	0.93 (0.91 - 0.94)
	NS3_Hi	0.59 (0.56 - 0.61)	0.71 (0.65 - 0.76)	0.79 (0.74 - 0.84)	0.81 (0.76 - 0.86)	0.87 (0.84 - 0.90)	0.90 (0.86 - 0.95)	0.91 (0.87 - 0.96)
	NS4B_Hi	0.58 (0.55 - 0.60)	0.72 (0.68 - 0.76)	0.77 (0.73 - 0.81)	0.81 (0.77 - 0.85)	0.84 (0.80 - 0.88)	0.88 (0.84 - 0.92)	0.88 (0.85 - 0.92)
	NS1_Low	0.53 (0.48 - 0.58)	0.63 (0.55 - 0.70)	0.71 (0.63 - 0.79)	0.80 (0.73 - 0.86)	0.91 (0.86 - 0.96)	0.93 (0.91 - 0.95)	0.94 (0.91 - 0.97)
	NS3_Low	0.54 (0.49 - 0.59)	0.67 (0.62 - 0.72)	0.76 (0.70 - 0.81)	0.79 (0.73 - 0.85)	0.85 (0.79 - 0.92)	0.88 (0.82 - 0.94)	0.89 (0.82 - 0.96)
	NS5_Low	0.51 (0.47 - 0.55)	0.57 (0.54 - 0.60)	0.59 (0.55 - 0.62)	0.66 (0.61 - 0.71)	0.73 (0.69 - 0.77)	0.74 (0.70 - 0.78)	0.73 (0.69 - 0.77)

Con_Hi	0.87 (0.83 - 0.91)	0.91 (0.87 - 0.94)	0.92 (0.89 - 0.95)	0.93 (0.91 - 0.96)
Con_Low	0.73 (0.69 - 0.76)	0.78 (0.72 - 0.84)	0.85 (0.79 - 0.91)	0.88 (0.83 - 0.93)

Table S2. Mean K-score values for ZIKV, YFV, WNV and DENV serotypes. The table shows the 95% confidence intervals of the mean for the analyzed regions across all evaluated segment lengths.

**Virus**	**Region**	**Mean (CI 95%)**						
		**300 nt length**	**500 nt length**	**700 nt length**	**900 nt length**	**1500 nt length**	**2100 nt length**	**2700 nt length**
ZIKV	C_Hi	0.014 (0.011 - 0.017)	0.013 (0.009 - 0.017)	0.011 (0.009 - 0.013)	0.010 (0.008 - 0.011)	0.008 (0.006 - 0.009)	0.006 (0.005 - 0.007)	0.005 (0.004 - 0.006)
	E_Hi	0.018 (0.011 - 0.025)	0.014 (0.011 - 0.017)	0.013 (0.009 - 0.017)	0.009 (0.007 - 0.011)	0.007 (0.005 - 0.008)	0.006 (0.005 - 0.007)	0.005 (0.004 - 0.006)
	NS3_Hi	0.016 (0.010 - 0.021)	0.011 (0.009 - 0.014)	0.009 (0.008 - 0.011)	0.009 (0.007 - 0.011)	0.007 (0.006 - 0.009)	0.006 (0.005 - 0.008)	0.004 (0.004 - 0.005)
	E_Low	0.016 (0.010 - 0.021)	0.013 (0.010 - 0.016)	0.010 (0.008 - 0.012)	0.009 (0.007 - 0.011)	0.007 (0.006 - 0.008)	0.006 (0.005 - 0.006)	0.005 (0.004 - 0.005)
	NS4B_Low	0.014 (0.010 - 0.017)	0.011 (0.009 - 0.014)	0.010 (0.008 - 0.013)	0.010 (0.008 - 0.011)	0.008 (0.007 - 0.009)	0.006 (0.005 - 0.007)	0.005 (0.004 - 0.005)
	NS5_Low	0.013 (0.012 - 0.015)	0.011 (0.011 - 0.012)	0.010 (0.009 - 0.011)	0.009 (0.008 - 0.011)	0.006 (0.005 - 0.008)	0.006 (0.005 - 0.007)	0.006 (0.005 - 0.007)
	Con_Hi				0.010 (0.008 - 0.013)	0.007 (0.006 - 0.008)	0.005 (0.005 - 0.006)	0.002 (0.002 - 0.003)
	Con_Low				0.008 (0.007 - 0.010)	0.007 (0.006 - 0.008)	0.005 (0.005 - 0.006)	0.003 (0.003 - 0.003)
YFV	NS1_Hi	0.188 (0.155 - 0.220)	0.097 (0.070 - 0.124)	0.073 (0.053- 0.093)	0.065 (0.050 - 0.079)	0.052 (0.042 - 0.061)	0.038 (0.029 - 0.047)	0.029 (0.020 - 0.038)
	NS3_Hi	0.145 (0.123 - 0.167)	0.102 (0.076 - 0.127)	0.098 (0.076 - 0.121)	0.068 (0.047 - 0.089)	0.048 (0.033 - 0.063)	0.035 (0.026 - 0.044)	0.031 (0.025 - 0.037)
	NS5_Hi	0.148 (0.129 - 0.167)	0.128 (0.107 - 0.148)	0.137 (0.117 - 0.157)	0.116 (0.098 - 0.134)	0.080 (0.063 - 0.097)	0.044 (0.028 - 0.061)	0.041 (0.028 - 0.054)
	E_Low	0.240 (0.201 - 0.279)	0.187 (0.154 - 0.220)	0.117 (0.096 - 0.137)	0.121 (0.100 - 0.141)	0.085 (0.069 - 0.101)	0.047 (0.038 - 0.057)	0.040 (0.033 - 0.046)
	NS3_Low	0.165 (0.143 - 0.187)	0.152 (0.130 - 0.173)	0.127 (0.102 - 0.151)	0.091 (0.063 - 0.120)	0.049 (0.040 - 0.058)	0.042 (0.032 - 0.052)	0.033 (0.025 - 0.041)
	NS5_Low	0.165 (0.138 - 0.192)	0.136 (0.101 - 0.171)	0.108 (0.085 - 0.131)	0.081 (0.059 - 0.103)	0.046 (0.028 - 0.064)	0.045 (0.030 - 0.059)	0.044 (0.029 - 0.060)
	Con_Hi				0.087 (0.074 - 0.100)	0.049 (0.037 - 0.060)	0.041 (0.031 - 0.051)	0.029 (0.021 - 0.037)
	Con_Low				0.106 (0.100 - 0.112)	0.075 (0.062 - 0.087)	0.050 (0.036 - 0.064)	0.035 (0.029 - 0.041)
WNV	NS2A_Hi	0.286 (0.230 - 0.342)	0.207 (0.181 - 0.232)	0.151 (0.100 - 0.202)	0.131 (0.088 - 0.173)	0.121 (0.094 - 0.148)	0.094 (0.064 - 0.123)	0.066 (0.053 - 0.080)
	NS3_Hi	0.254 (0.212 - 0.296)	0.140 (0.114 - 0.165)	0.137 (0.111 - 0.163)	0.109 (0.097 - 0.122)	0.103 (0.070 - 0.135)	0.106 (0.070 - 0.142)	0.078 (0.061 - 0.095)
	NS4B_Hi	0.254 (0.213 - 0.294)	0.228 (0.177 - 0.278)	0.176 (0.122 - 0.229)	0.158 (0.125 - 0.192)	0.108 (0.095 - 0.121)	0.098 (0.083 - 0.114)	0.082 (0.064 - 0.101)
	E_Low	0.229 (0.192 - 0.266)	0.178 (0.157 - 0.199)	0.140 (0.106 - 0.174)	0.120 (0.105 - 0.134)	0.131 (0.118 - 0.143)	0.069 (0.056 - 0.083)	0.072 (0.068 - 0.077)
	NS1_Low	0.285 (0.222 - 0.347)	0.208 (0.164 - 0.251)	0.208 (0.145 - 0.271)	0.131 (0.094 - 0.168)	0.080 (0.057 - 0.102)	0.087 (0.071 - 0.104)	0.063 (0.044 - 0.082)
	NS5_Low	0.264 (0.224 - 0.305)	0.312 (0.240 - 0.383)	0.259 (0.183 - 0.335)	0.177 (0.114 - 0.239)	0.167 (0.101 - 0.233)	0.169 (0.103 - 0.234)	0.168 (0.102 - 0.235)
	Con_Hi				0.169 (0.124 - 0.215)	0.119 (0.086 - 0.152)	0.098 (0.060 - 0.136)	0.073 (0.061 - 0.085)
	Con_Low				0.175 (0.143 - 0.207)	0.147 (0.118 - 0.175)	0.121 (0.091 - 0.151)	0.082 (0.066 - 0.099)
DENV-1	E_Hi	0.046 (0.036 - 0.057)	0.041 (0.031 - 0.051)	0.037 (0.028 - 0.046)	0.034 (0.026 - 0.042)	0.027 (0.025 - 0.029)	0.024 (0.020 - 0.027)	0.020 (0.016 - 0.025)
	NS2A_Hi	0.051 (0.041 - 0.061)	0.047 (0.035 - 0.058)	0.041 (0.029 - 0.053)	0.036 (0.024 - 0.048)	0.032 (0.020 - 0.043)	0.032 (0.019 - 0.045)	0.027 (0.015 - 0.038)
	NS4A_Hi	0.049 (0.043 - 0.055)	0.043 (0.039 - 0.048)	0.036 (0.032 - 0.041)	0.033 (0.031 - 0.035)	0.027 (0.024 - 0.030)	0.023 (0.020 - 0.026)	0.019 (0.016 - 0.023)
	C_Low	0.063 (0.054 - 0.071)	0.048 (0.035 - 0.061)	0.048 (0.034 - 0.063)	0.047 (0.030 - 0.063)	0.037 (0.019 - 0.055)	0.028 (0.011 - 0.045)	0.022 (0.007 - 0.038)

	N3_Low	0.054 (0.048 - 0.060)	0.048 (0.041 - 0.056)	0.048 (0.040 - 0.056)	0.046 (0.036 - 0.056)	0.039 (0.025 - 0.054)	0.030 (0.024 - 0.035)	0.024 (0.018 - 0.029)
	NS5_Low	0.059 (0.050 - 0.067)	0.047 (0.040 - 0.054)	0.038 (0.033 - 0.043)	0.032 (0.029 - 0.035)	0.030 (0.026 - 0.034)	0.030 (0.026 - 0.035)	0.029 (0.025 - 0.033)
	Con_Hi				0.033 (0.027 - 0.038)	0.029 (0.021 - 0.037)	0.022 (0.019 - 0.025)	0.018 (0.016 - 0.021)
	Con_Low				0.040 (0.031 - 0.049)	0.033 (0.021 - 0.045)	0.030 (0.018 - 0.043)	0.030 (0.016 - 0.043)
DENV-2	NS2A_Hi	0.102 (0.075 - 0.130)	0.090 (0.052 - 0.128)	0.090 (0.059 - 0.122)	0.088 (0.056 - 0.120)	0.063 (0.042 - 0.084)	0.060 (0.041 - 0.078)	0.050 (0.027 - 0.073)
	NS3_Hi	0.164 (0.113 - 0.215)	0.137 (0.093 - 0.180)	0.125 (0.086 - 0.164)	0.117 (0.086 - 0.148)	0.092 (0.063 - 0.121)	0.075 (0.045 - 0.104)	0.067 (0.037 - 0.097)
	NS4A_Hi	0.142 (0.107 - 0.176)	0.133 (0.102 - 0.164)	0.110 (0.074 - 0.146)	0.091 (0.058 - 0.124)	0.076 (0.045 - 0.107)	0.071 (0.037 - 0.106)	0.061 (0.027 - 0.095)
	M_Low	0.118 (0.068 - 0.167)	0.106 (0.054 - 0.159)	0.093 (0.055 - 0.131)	0.082 (0.053 - 0.112)	0.071 (0.051 - 0.090)	0.052 (0.026 - 0.078)	0.054 (0.030 - 0.078)
	NS3_Low	0.188 (0.115 - 0.261)	0.139 (0.088 - 0.190)	0.120 (0.083 - 0.158)	0.106 (0.069 - 0.143)	0.079 (0.053 - 0.106)	0.061 (0.029 - 0.092)	0.063 (0.030 - 0.095)
	NS5_Low	0.161 (0.118 - 0.204)	0.098 (0.073 - 0.124)	0.096 (0.072 - 0.120)	0.091 (0.059 - 0.122)	0.079 (0.041 - 0.117)	0.080 (0.042 - 0.117)	0.079 (0.041 - 0.117)
	Con_Hi				0.080 (0.056 - 0.104)	0.068 (0.051 - 0.085)	0.063 (0.045 - 0.082)	0.055 (0.038 - 0.071)
	Con_Low				0.062 (0.045 - 0.079)	0.046 (0.037 - 0.056)	0.042 (0.032 - 0.053)	0.035 (0.026 - 0.044)
DENV-3	E_Hi	0.042 (0.036 - 0.048)	0.035 (0.030 - 0.040)	0.030 (0.026 - 0.033)	0.024 (0.020 - 0.028)	0.023 (0.017 - 0.029)	0.017 (0.012 - 0.022)	0.016 (0.011 - 0.020)
	NS2A_Hi	0.040 (0.033 - 0.046)	0.031 (0.026 - 0.035)	0.027 (0.023 - 0.030)	0.022 (0.017 - 0.028)	0.020 (0.016 - 0.024)	0.018 (0.014 - 0.021)	0.016 (0.013 - 0.018)
	NS4B_Hi	0.038 (0.035 - 0.041)	0.031 (0.029 - 0.032)	0.026 (0.024 - 0.027)	0.021 (0.020 - 0.022)	0.020 (0.017 - 0.022)	0.017 (0.013 - 0.022)	0.016 (0.011 - 0.021)
	C_Low	0.038 (0.037 - 0.040)	0.034 (0.031 - 0.037)	0.028 (0.026 - 0.031)	0.027 (0.023 - 0.031)	0.022 (0.016 - 0.029)	0.019 (0.013 - 0.025)	0.016 (0.011 - 0.022)
	NS1_Low	0.046 (0.042 - 0.050)	0.043 (0.037 - 0.048)	0.038 (0.032 - 0.043)	0.031 (0.026 - 0.037)	0.021 (0.018 - 0.025)	0.019 (0.016 - 0.022)	0.021 (0.015 - 0.027)
	NS5_Low	0.051 (0.056 - 0.046)	0.038 (0.034 - 0.042)	0.034 (0.032 - 0.036)	0.031 (0.028 - 0.033)	0.031 (0.028 - 0.033)	0.031 (0.028 - 0.033)	0.031 (0.028 - 0.033)
	Con_Hi				0.024 (0.022 - 0.026)	0.020 (0.018 - 0.022)	0.017 (0.016 - 0.019)	0.014 (0.012 - 0.016)
	Con_Low				0.025 (0.021 - 0.028)	0.021 (0.018 - 0.025)	0.020 (0.016 - 0.023)	0.017 (0.014 - 0.020)
DENV-4	NS2A_Hi	0.056 (0.044 - 0.068)	0.049 (0.032 - 0.065)	0.043 (0.027 - 0.059)	0.043 (0.029 - 0.056)	0.037 (0.022 - 0.051)	0.028 (0.016 - 0.041)	0.027 (0.015 - 0.039)
	NS3_Hi	0.055 (0.046 - 0.063)	0.050 (0.038 - 0.061)	0.039 (0.028 - 0.050)	0.033 (0.025 - 0.040)	0.025 (0.017 - 0.033)	0.025 (0.010 - 0.039)	0.018 (0.010 - 0.025)
	NS4B_Hi	0.050 (0.045 - 0.056)	0.037 (0.026 - 0.047)	0.039 (0.028 - 0.050)	0.035 (0.023 - 0.047)	0.033 (0.022 - 0.044)	0.026 (0.021 - 0.031)	0.022 (0.018 - 0.026)
	NS1_Low	0.057 (0.046 - 0.067)	0.045 (0.035 - 0.054)	0.036 (0.030 - 0.041)	0.035 (0.029 - 0.040)	0.030 (0.023 - 0.037)	0.027 (0.020 - 0.034)	0.024 (0.018 - 0.031)
	NS3_Low	0.062 (0.047 - 0.077)	0.044 (0.029 - 0.060)	0.042 (0.021 - 0.063)	0.038 (0.022 - 0.054)	0.026 (0.015 - 0.036)	0.019 (0.015 - 0.024)	0.016 (0.014 - 0.019)
	NS5_Low	0.055 (0.050 - 0.061)	0.043 (0.032 - 0.054)	0.040 (0.031 - 0.050)	0.039 (0.030 - 0.047)	0.037 (0.030 - 0.045)	0.037 (0.030 - 0.045)	0.037 (0.029 - 0.045)
	Con_Hi				0.031 (0.02 - 0.04)		0.022 (0.01 - 0.03)	0.020 (0.01 - 0.03)
	Con_Low				0.036 (0.03 - 0.04)		0.024 (0.02 - 0.03)	0.021 (0.01 - 0.03)

Table S3. Mean Scale Factor values for ZIKV, YFV, WNV and DENV serotypes. The table shows the 95% confidence intervals of the mean for the analyzed regions across all evaluated segment lengths.

Virus	Region	Mean (CI 95%)						
		**300 nt length**	**500 nt length**	**700 nt length**	**900 nt length**	**1500 nt length**	**2100 nt length**	**2700 nt length**
ZIKV	C_Hi	1.35 (1.06 - 1.64)	1.26 (1.02 - 1.51)	1.16 (1.00 - 1.32)	1.19 (1.08 - 1.31)	1.19 (1.10 - 1.28)	1.11 (1.06 - 1.16)	1.03 (0.98 - 1.07)
	E_Hi	1.31 (0.93 - 1.70)	1.19 (0.96 - 1.42)	1.25 (1.01 - 1.50)	1.09 (0.97 - 1.22)	1.11 (0.98 - 1.23)	1.04 (0.94 - 1.14)	1.05 (0.96 - 1.15)
	NS3_Hi	1.84 (1.43 - 2.25)	1.32 (1.22 - 1.41)	1.28 (1.14 - 1.43)	1.28 (1.13 - 1.44)	1.39 (1.22 - 1.56)	1.21 (1.10 - 1.32)	1.12 (1.09 - 1.15)
	E_Low	2.10 (1.74 - 2.45)	1.67 (1.39 - 1.95)	1.47 (1.28 - 1.65)	1.42 (1.26 - 1.58)	1.17 (1.06 - 1.29)	1.07 (1.00 - 1.14)	1.06 (0.97 - 1.16)
	NS4B_Low	2.12 (1.86 - 2.37)	1.92 (1.64 - 2.21)	1.63 (1.52 - 1.73)	1.63 (1.53 - 1.73)	1.33 (1.13 - 1.53)	1.18 (1.06 - 1.31)	1.13 (1.04 - 1.23)
	NS5_Low	1.88 (1.51 - 2.25)	1.57 (1.25 - 1.88)	1.41 (1.28 - 1.55)	1.29 (1.20 - 1.39)	1.07 (1.00 - 1.14)	1.06 (1.00 - 1.11)	1.05 (1.00 - 1.11)
	Con_Hi				1.13 (0.96 - 1.30)	1.02 (0.95 - 1.09)	0.98 (0.94 - 1.02)	0.98 (0.95 - 1.01)
	Con_Low				1.51 (1.46 - 1.55)	1.27 (1.13 - 1.41)	1.17 (1.10 - 1.23)	0.97 (0.94 - 1.00)
YFV	NS1_Hi	2.07 (1.78 - 2.36)	1.68 (1.44 - 1.92)	1.59 (1.37 - 1.81)	1.48 (1.28 - 1.69)	1.25 (1.09 - 1.40)	1.06 (0.94 - 1.18)	1.07 (0.98 - 1.17)
	NS3_Hi	1.92 (1.63 - 2.22)	1.67 (1.45 - 1.89)	1.39 (1.19 - 1.59)	1.27 (1.12 - 1.43)	1.24 (1.13 - 1.35)	1.09 (1.00 - 1.17)	1.03 (0.98 - 1.09)
	NS5_Hi	2.11 (1.83 - 2.39)	1.87 (1.65 - 2.09)	1.68 (1.51 - 1.86)	1.72 (1.57 - 1.87)	1.50 (1.37 - 1.64)	1.33 (1.20 - 1.47)	1.32 (1.20 - 1.44)
	E_Low	3.01 (2.59 - 3.43)	2.34 (2.09 - 2.58)	2.06 (1.83 - 2.29)	2.06 (1.81 - 2.31)	1.53 (1.36 - 1.70)	1.34 (1.20 - 1.49)	1.18 (1.06 - 1.30)
	NS3_Low	3.34 (2.71 - 3.96)	2.41 (2.00 - 2.81)	2.00 (1.73 - 2.28)	1.72 (1.51 - 1.93)	1.29 (1.15 - 1.44)	1.27 (1.19 - 1.36)	1.23 (1.15 - 1.32)
	NS5_Low	3.10 (2.72 - 3.48)	2.24 (2.00 - 2.47)	2.01 (1.84 - 2.18)	1.73 (1.57 - 1.88)	1.52 (1.37 - 1.67)	1.50 (1.37 - 1.63)	1.50 (1.36 - 1.64)
	Con_Hi				1.38 (1.20 - 1.57)	1.19 (1.04 - 1.34)	1.08 (0.95 - 1.21)	1.05 (0.95 - 1.16)
	Con_Low				2.13 (1.90 - 2.35)	1.69 (1.49 - 1.89)	1.46 (1.33 - 1.60)	1.32 (1.19 - 1.45)
WNV	NS2A_Hi	1.57 (1.49 - 1.65)	1.33 (1.21 - 1.44)	1.24 (1.15 - 1.32)	1.26 (1.21 - 1.32)	1.21 (1.13 - 1.29)	1.13 (1.07 - 1.18)	1.15 (1.11 - 1.20)
	NS3_Hi	1.84 (1.74 - 1.95)	1.50 (1.45 - 1.55)	1.31 (1.27 - 1.35)	1.15 (1.13 - 1.18)	1.12 (1.10 - 1.14)	1.14 (1.07 - 1.21)	1.12 (1.09 - 1.15)
	NS4B_Hi	1.90 (1.74 - 2.06)	1.72 (1.62 - 1.81)	1.75 (1.62 - 1.89)	1.64 (1.52 - 1.77)	1.42 (1.34 - 1.49)	1.36 (1.26 - 1.45)	1.33 (1.23 - 1.43)
	E_Low	2.08 (1.85 - 2.31)	1.81 (1.66 - 1.95)	1.57 (1.52 - 1.62)	1.45 (1.38 - 1.51)	1.27 (1.20 - 1.34)	1.13 (1.07 - 1.19)	1.13 (1.07 - 1.18)
	NS1_Low	1.85 (1.76 - 1.93)	2.03 (1.89 - 2.17)	1.62 (1.54 - 1.70)	1.38 (1.30 - 1.46)	1.29 (1.21 - 1.37)	1.17 (1.08 - 1.25)	1.16 (1.10 - 1.22)
	NS5_Low	2.34 (2.16 - 2.52)	2.14 (2.05 - 2.23)	1.85 (1.78 - 1.92)	1.66 (1.56 - 1.77)	1.56 (1.46 - 1.66)	1.56 (1.45 - 1.66)	1.56 (1.46 - 1.66)
	Con_Hi				1.27 (1.19 - 1.36)	1.11 (1.04 - 1.18)	1.08 (1.01 - 1.16)	1.05 (0.98 - 1.11)
	Con_Low				1.46 (1.34 - 1.59)	1.46 (1.37 - 1.55)	1.28 (1.23 - 1.33)	1.19 (1.12 - 1.26)
DENV-1	E_Hi	0.97 (0.92 - 1.02)	1.03 (0.97 - 1.09)	0.89 (0.86 - 0.93)	0.96 (0.94 - 0.98)	0.97 (0.95 - 0.99)	0.90 (0.89 - 0.92)	0.91 (0.90 - 0.93)
	NS2A_Hi	0.86 (0.82 - 0.91)	0.71 (0.66 - 0.75)	0.74 (0.71 - 0.77)	0.80 (0.76 - 0.84)	0.84 (0.81 - 0.88)	0.88 (0.82 - 0.94)	0.92 (0.88 - 0.95)
	NS4A_Hi	0.89 (0.71 - 1.06)	0.84 (0.73 - 0.95)	0.85 (0.78 - 0.91)	0.88 (0.84 - 0.92)	0.91 (0.88 - 0.94)	0.98 (0.97 - 0.99)	1.01 (0.98 - 1.03)
	C_Low	1.25 (1.15 - 1.35)	1.10 (1.03 - 1.17)	1.05 (0.98 - 1.13)	1.02 (0.95 - 1.09)	0.96 (0.88 - 1.03)	0.97 (0.90 - 1.04)	0.97 (0.92 - 1.02)
	N3_Low	1.06 (1.00 - 1.12)	0.99 (0.91 - 1.07)	1.01 (0.93 - 1.09)	0.98 (0.91 - 1.04)	1.00 (0.89 - 1.11)	0.98 (0.96 - 1.01)	0.99 (0.96 - 1.01)
	NS5_Low	1.26 (1.21 - 1.32)	1.26 (1.23 - 1.29)	1.20 (1.16 - 1.25)	1.21 (1.18 - 1.25)	1.06 (1.03 - 1.10)	1.06 (1.03 - 1.10)	1.06 (1.03 - 1.10)
	Con_Hi				0.90 (0.86 - 0.94)	0.86 (0.83 - 0.88)	0.85 (0.82 - 0.87)	0.90 (0.88 - 0.92)
	Con_Low				1.19 (1.14 - 1.25)	1.13 (1.07 - 1.19)	1.11 (1.06 - 1.17)	1.08 (1.02 - 1.14)
DENV-2	NS2A_Hi	1.25 (1.08 - 1.43)	1.31 (1.07 - 1.54)	1.18 (0.92 - 1.45)	1.13 (0.82 - 1.45)	1.02 (0.73 - 1.30)	0.96 (0.66 - 1.26)	0.92 (0.63 - 1.22)
	NS3_Hi	1.91 (1.22 - 2.60)	1.54 (1.08 - 2.00)	1.43 (1.09 - 1.77)	1.36 (1.07 - 1.66)	1.29 (1.09 - 1.50)	1.20 (1.09 - 1.31)	1.12 (1.05 - 1.19)
	NS4A_Hi	1.23 (0.80 - 1.66)	1.14 (0.82 - 1.46)	1.14 (0.94 - 1.35)	1.11 (0.96 - 1.25)	1.10 (0.91 - 1.30)	1.07 (0.86 - 1.28)	1.05 (0.93 - 1.18)
	M_Low	1.53 (0.81 - 2.25)	1.50 (0.72 - 2.27)	1.38 (0.72 - 2.05)	1.23 (0.69 - 1.76)	1.05 (0.63 - 1.46)	0.99 (0.63 - 1.34)	0.94 (0.61 - 1.26)
	NS3_Low	1.68 (0.92 - 2.44)	1.48 (0.99 - 1.98)	1.46 (1.08 - 1.84)	1.42 (1.12 - 1.72)	1.16 (1.05 - 1.27)	1.13 (1.06 - 1.21)	1.08 (0.94 - 1.22)
	NS5_Low	2.09 (1.50 - 2.68)	1.62 (1.36 - 1.88)	1.69 (1.42 - 1.96)	1.54 (1.27 - 1.81)	1.29 (1.09 - 1.50)	1.30 (1.11 - 1.50)	1.30 (1.10 - 1.49)
	Con_Hi				1.07 (0.96 - 1.19)	1.11 (1.05 - 1.17)	1.09 (1.02 - 1.16)	1.09 (1.03 - 1.15)
	Con_Low				1.36 (1.14 - 1.58)	1.22 (1.08 - 1.36)	1.23 (1.11 - 1.35)	1.19 (1.09 - 1.30)
DENV-3	E_Hi	0.99 (0.89 - 1.08)	0.93 (0.88 - 0.98)	0.93 (0.90 - 0.96)	0.95 (0.92 - 0.98)	0.94 (0.90 - 0.98)	0.99 (0.96 - 1.02)	1.01 (0.98 - 1.05)
	NS2A_Hi	0.95 (0.86 - 1.04)	0.90 (0.84 - 0.96)	0.93 (0.90 - 0.97)	0.95 (0.90 - 1.00)	0.98 (0.95 - 1.02)	0.98 (0.93 - 1.03)	0.98 (0.94 - 1.03)
	NS4B_Hi	0.91 (0.83 - 0.99)	1.01 (0.95 - 1.08)	1.00 (0.93 - 1.07)	1.00 (0.95 - 1.04)	1.00 (0.97 - 1.03)	0.99 (0.97 - 1.02)	0.98 (0.94 - 1.02)
	C_Low	1.43 (1.30 - 1.55)	1.39 (1.28 - 1.49)	1.32 (1.24 - 1.39)	1.22 (1.16 - 1.28)	1.06 (1.03 - 1.09)	1.03 (1.00 - 1.05)	1.02 (1.00 - 1.04)
	NS1_Low	1.30 (1.18 - 1.41)	1.10 (1.02 - 1.18)	1.16 (1.08 - 1.23)	1.16 (1.09 - 1.24)	1.02 (0.96 - 1.08)	1.03 (0.99 - 1.07)	1.01 (0.95 - 1.07)
	NS5_Low	1.10 (0.81 - 1.38)	1.10 (0.92 - 1.27)	0.97 (0.91 - 1.03)	0.95 (0.90 - 1.00)	0.95 (0.90 - 1.01)	0.95 (0.90 - 1.01)	0.96 (0.91 - 1.01)
	Con_Hi				0.91 (0.87 - 0.94)	0.90 (0.88 - 0.92)	0.91 (0.90 - 0.92)	0.92 (0.91 - 0.94)
	Con_Low				1.19 (1.07 - 1.31)	1.17 (1.11 - 1.23)	1.11 (1.07 - 1.16)	1.09 (1.05 - 1.13)
DENV-4	NS2A_Hi	0.84 (0.72 - 0.97)	0.87 (0.76 - 0.98)	0.88 (0.79 - 0.98)	0.90 (0.83 - 0.98)	0.93 (0.87 - 0.99)	0.93 (0.88 - 0.97)	0.98 (0.93 - 1.03)
	NS3_Hi	1.27 (1.15 - 1.40)	1.11 (0.99 - 1.22)	1.06 (1.01 - 1.11)	1.04 (0.99 - 1.10)	1.04 (0.99 - 1.09)	1.03 (0.97 - 1.09)	1.00 (0.97 - 1.03)
	NS4B_Hi	1.00 (0.93 - 1.06)	1.03 (0.97 - 1.09)	1.02 (0.94 - 1.09)	1.01 (0.94 - 1.08)	1.01 (0.94 - 1.08)	1.04 (0.98 - 1.09)	1.03 (1.00 - 1.07)
	NS1_Low	1.22 (1.11 - 1.33)	1.07 (0.97 - 1.16)	1.14 (1.07 - 1.20)	1.12 (1.06 - 1.18)	0.97 (0.89 - 1.05)	0.98 (0.93 - 1.02)	0.97 (0.93 - 1.00)
	NS3_Low	1.45 (1.28 - 1.63)	1.26 (1.13 - 1.40)	1.26 (1.09 - 1.43)	1.22 (1.09 - 1.34)	1.04 (0.98 - 1.11)	1.04 (1.00 - 1.07)	1.02 (1.00 - 1.05)
	NS5_Low	1.47 (1.31 - 1.62)	1.14 (0.96 - 1.32)	1.14 (1.00 - 1.28)	1.10 (0.99 - 1.22)	1.09 (0.99 - 1.19)	1.09 (0.99 - 1.20)	1.09 (0.99 - 1.20)
	Con_Hi				0.91 (0.83 - 0.98)	0.92 (0.87 - 0.98)	0.92 (0.88 - 0.96)	0.93 (0.89 - 0.97)
	Con_Low				1.19 (1.11 - 1.28)	1.02 (0.93 - 1.11)	1.09 (1.01 - 1.18)	1.09 (1.03 - 1.15)

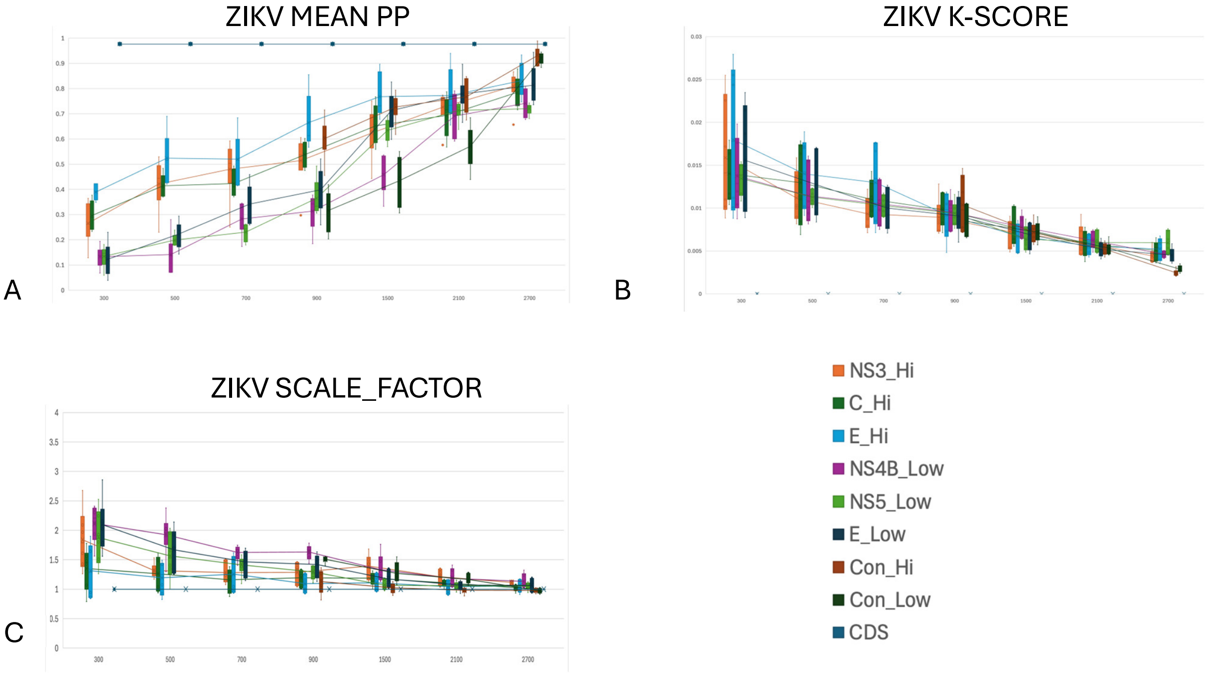

Figure S1. A-C. Phylogenetic analysis of ZIKV: A) Tree support analysis. B) Tree topological incongruence analysis. C) Tree branch length analysis. X-axis: length of the evaluated genomic regions. Y-axis: A. Average PP of tree nodes. B. K-score. C. Scale Factor. Across all evaluated regions, all parameters generally improved with increasing region length. The 2700 nt concatenated high-variability regions exhibited the most robust performance across all three metrics.

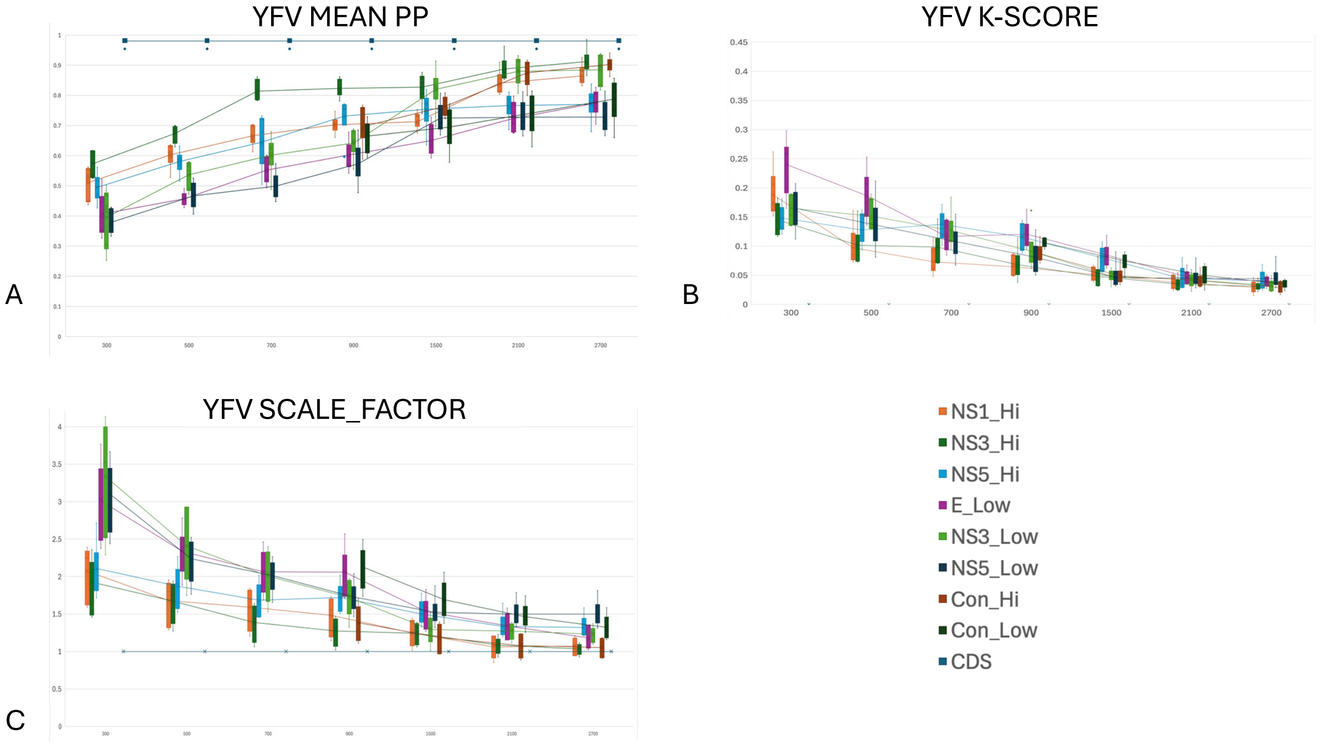

Figure S2. A-C Phylogenetic analysis of YFV: A) Tree support analysis. B) Topological incongruence analysis. C) Branch length divergence analysis. X-axis: length of the evaluated genomic regions. Y-

axis: A) Average PP of tree nodes. B) K-score. C) Scale Factor. Across all evaluated regions, all parameters generally improved with increasing region length. The 2700 nt high-variability region of NS3 exhibited the most robust performance across all three metrics.

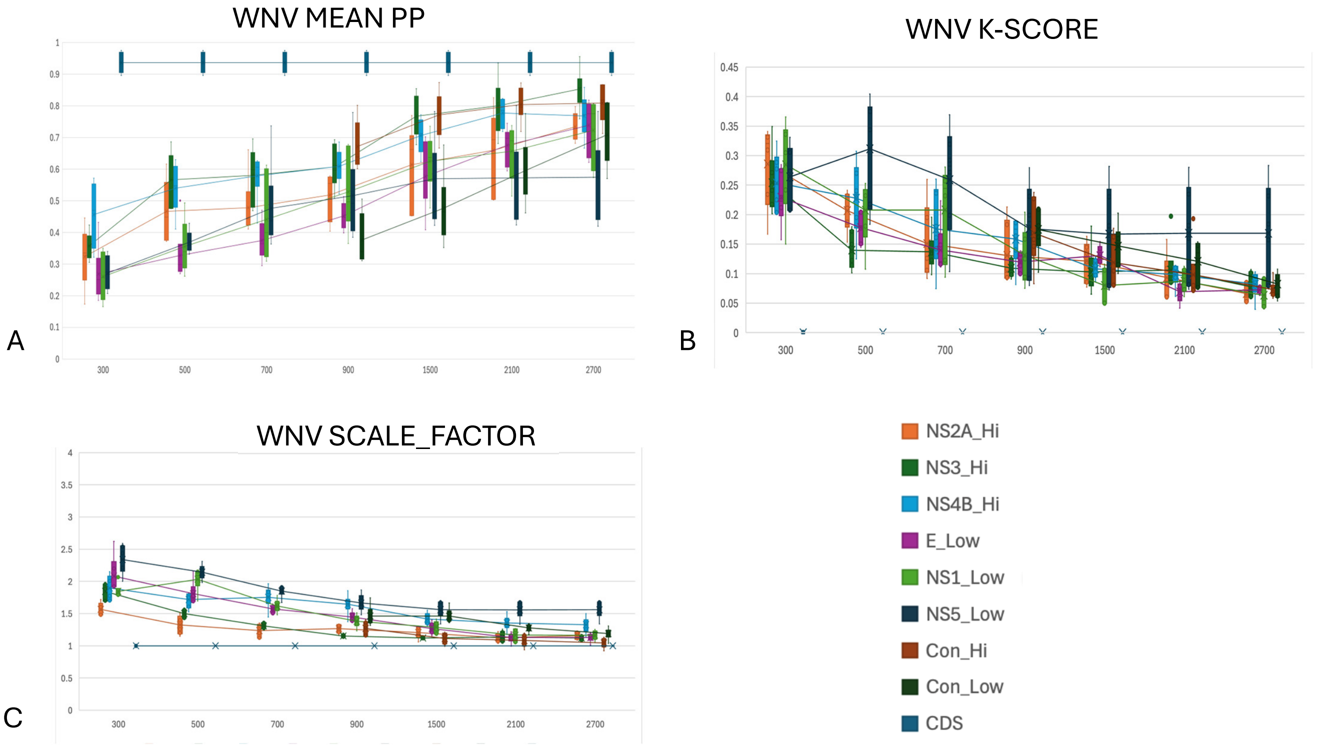

Figure S3. A-C. Phylogenetic analysis of WNV: A) Tree support analysis. B) Topological incongruence analysis. C) Branch length divergence analysis. X-axis: length of the evaluated genomic regions. Y-axis: A) Average PP of tree nodes. B) K-score. C) Scale Factor. Across all evaluated regions, all parameters generally improved with increasing region length. The 2700 nt catenated high-variability regions exhibited the most robust performance across all three metrics.

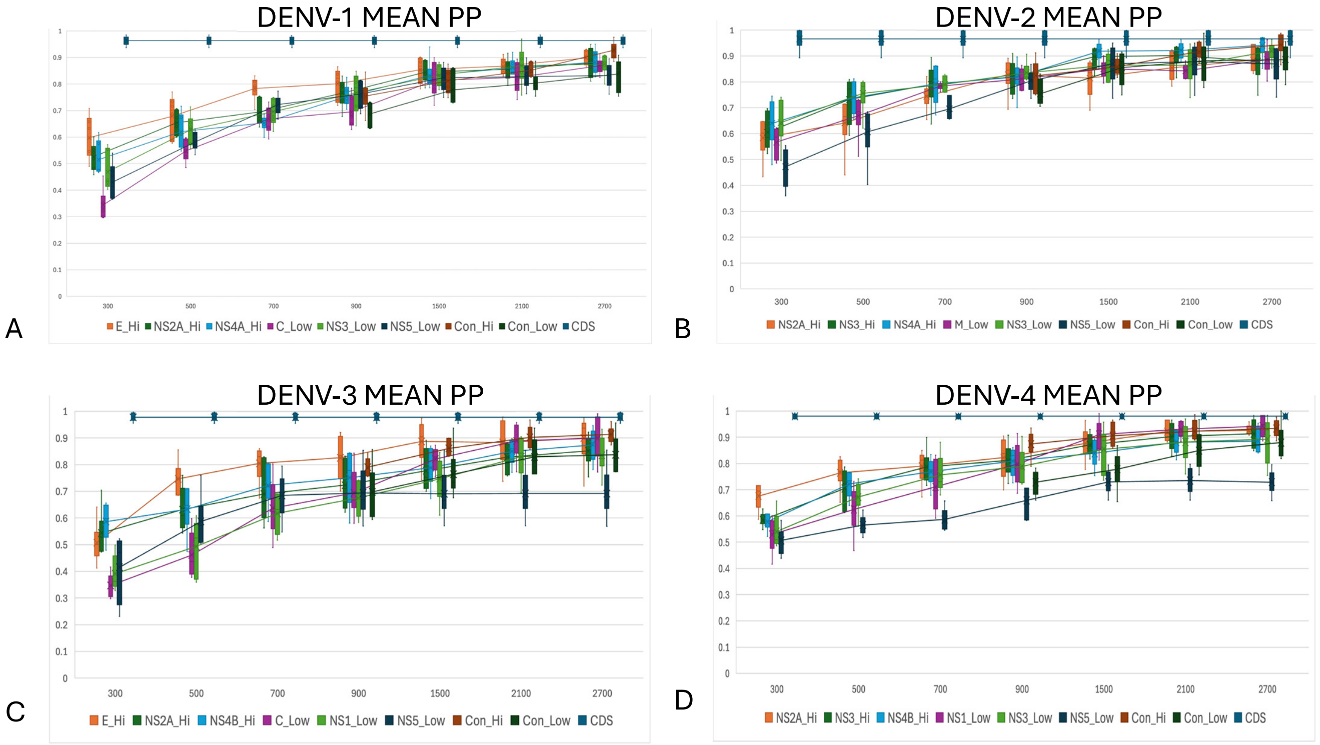

Figure S4. A–D. Tree Support Analysis for DENV Serotypes: A) DENV-1. B) DENV-2. C) DENV-3. D) DENV-4. Y-axis: average PP of tree nodes. X-axis: different lengths of evaluated regions. Across all evaluated regions, mean PP values generally improved with increasing region length. The best-performing regions for each serotype were 2700 nt high-variability regions: NS2A for DENV-3/4, NS4A for DENV-2, and E for DENV-1.

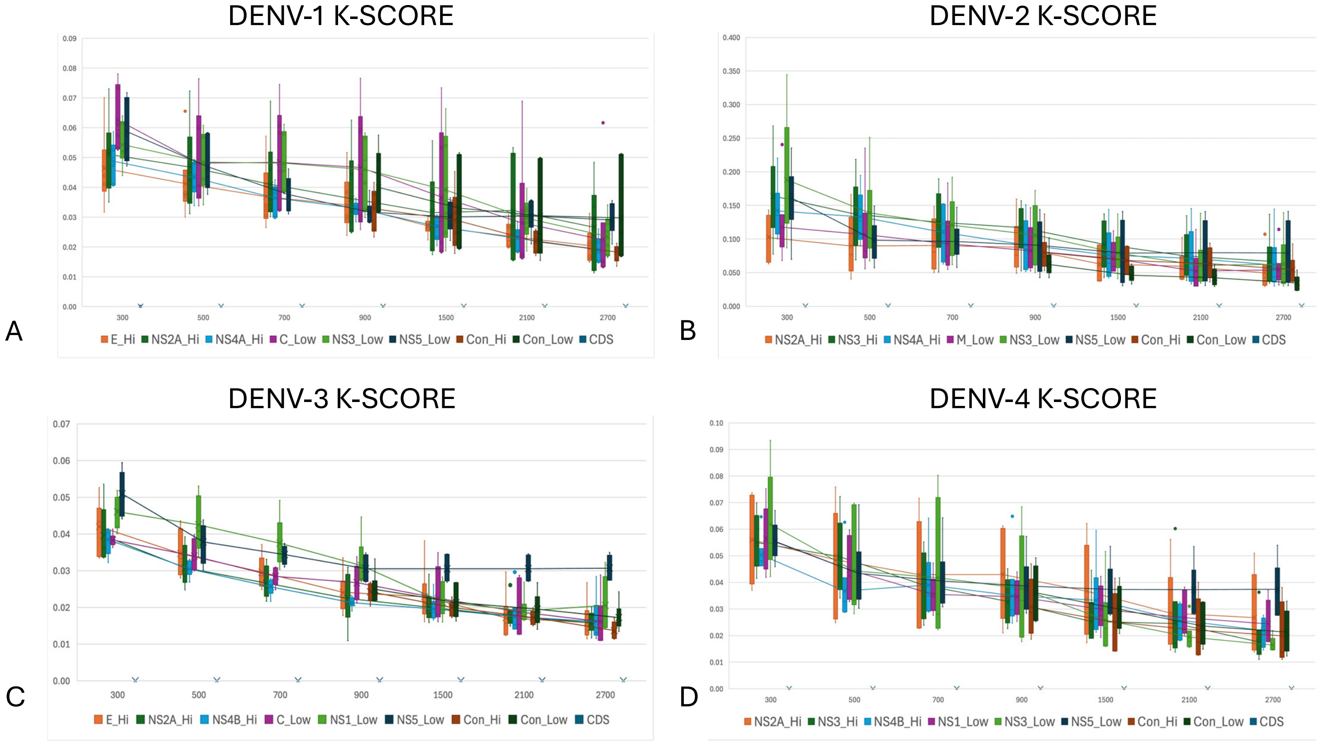

Figure S5. A–D. Topological Incongruence Analysis of Phylogenetic Trees for DENV Serotypes: A) DENV-1. B) DENV-2. C) DENV-3. D) DENV-4. Y-axis: K-score. X-axis: different lengths of evaluated

regions. Across all evaluated regions, K-score values decreased with increasing region length. The best-performing regions for each serotype were 2700 nt in length and included: the high-variability NS4A region for DENV-1; concatenated high-variability regions for DENV-2; the high-variability E region for DENV-3; and the high-variability NS3 region for DENV-4.

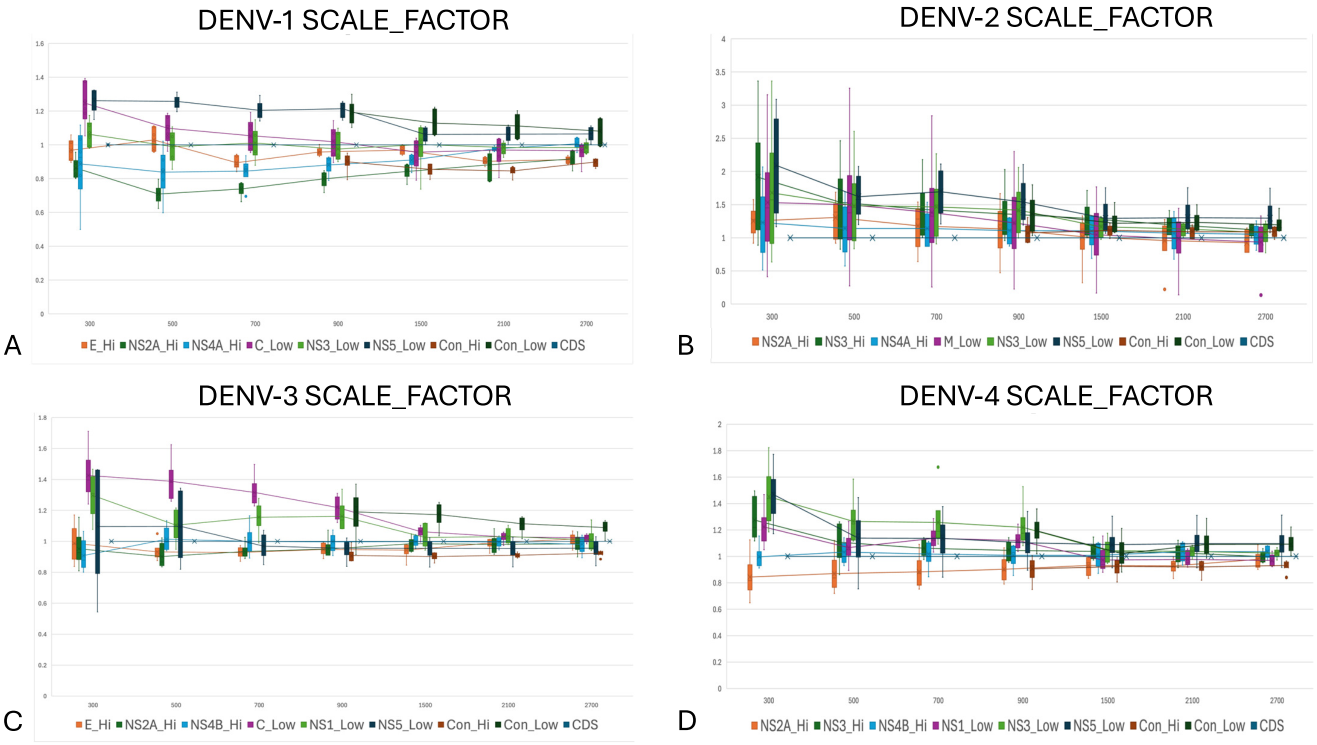

Figure S6. A–D Branch Length Analysis of Phylogenetic Trees for DENV Serotypes: Y-axis: Scale Factor. X-axis: different lengths of evaluated regions. A) DENV serotype 1. B) DENV serotype 2. C) DENV serotype 3. D) DENV serotype 4. Across all evaluated regions, Scale Factor values decreased with increasing region length. The best-performing regions for each serotype were were 2700 nt high-variability regions: NS4A region for DENV-1; concatenated regions for DENV-2; E region for DENV-3; and NS3 region for DENV-4.

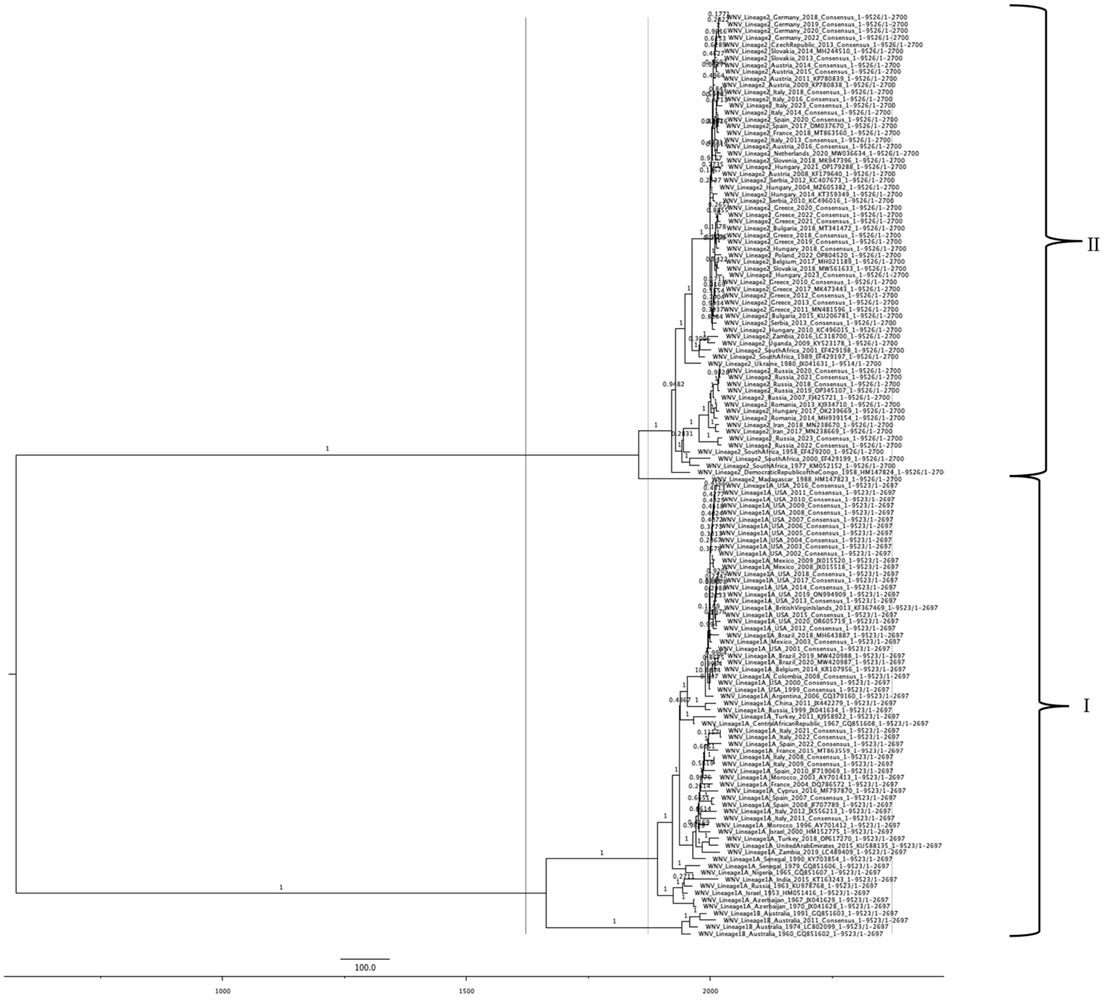

Figure S7. Maximum clade credibility phylogenetic tree of WNV, constructed with 136 sequences from 43 different countries. The corresponding lineages are indicated on each side of the tree: lineage I (I) and lineage II (II).

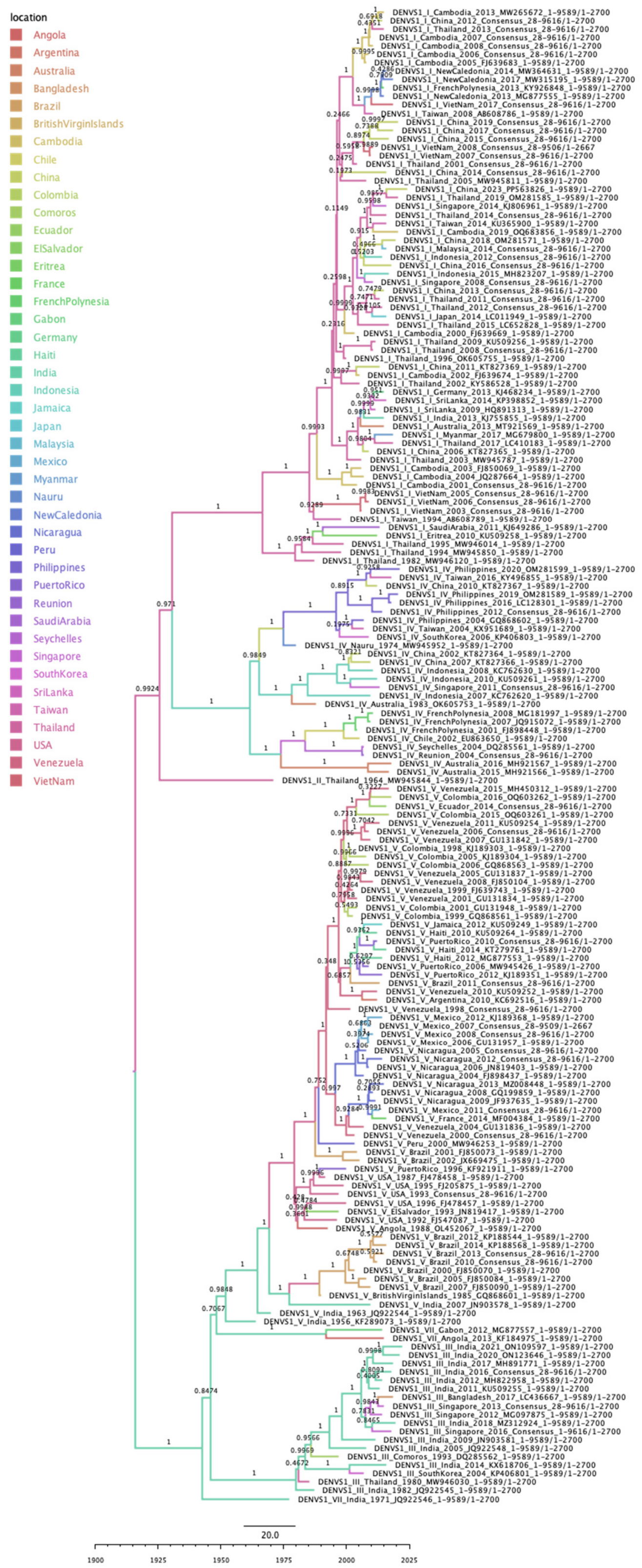

Figure S8. Maximum clade credibility phylogenetic tree of DENV-1, constructed with 177 sequences from 42 different countries. The tree is color-coded according to the geographic origin of the analyzed sequences.

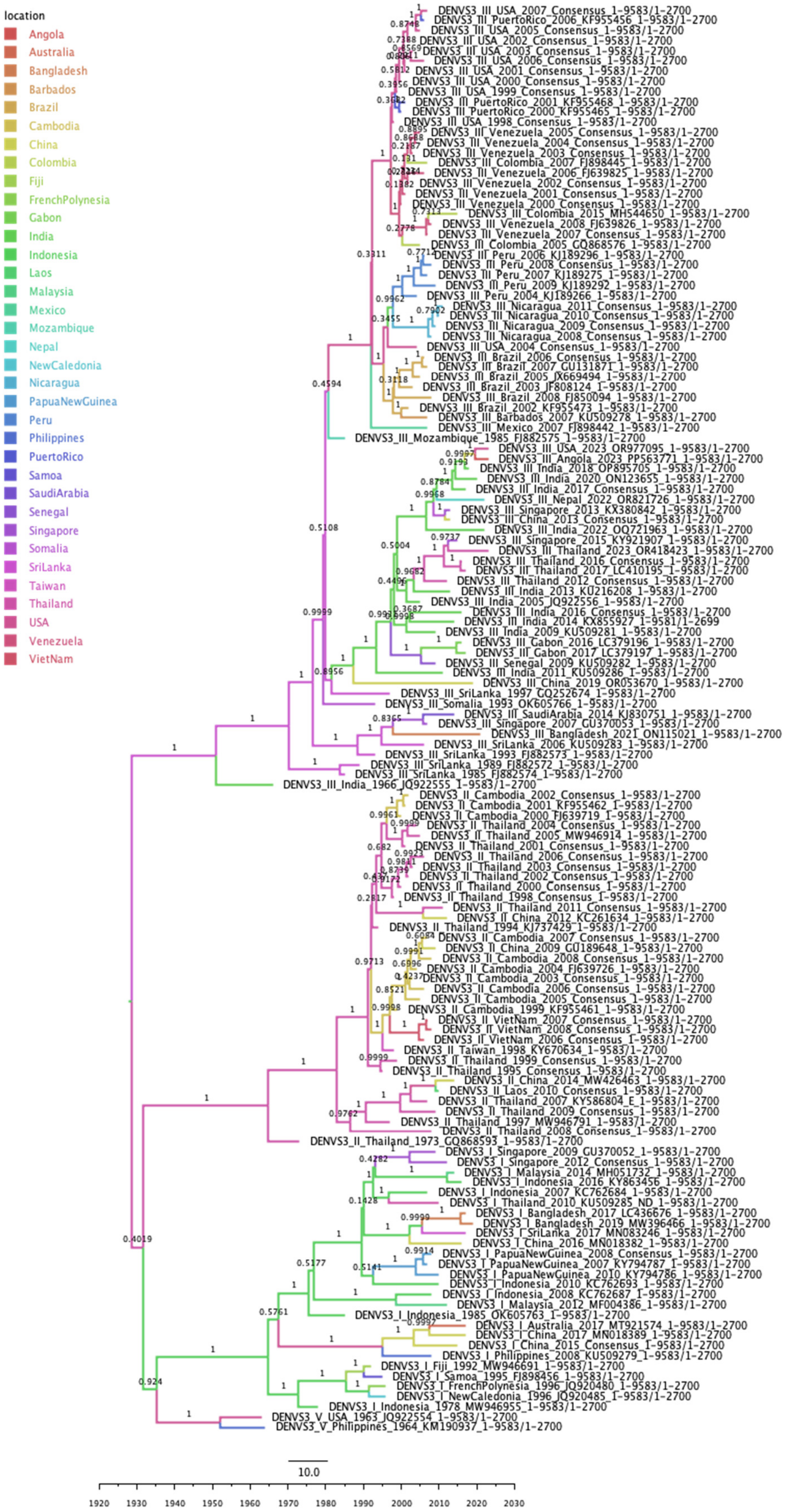

Figure S9. Maximum clade credibility phylogenetic tree of DENV-3, constructed with 140 sequences from 35 different countries. The tree is color-coded according to the geographic origin of the analyzed sequences.
